# Single-cell profiling identifies impaired adaptive NK cells expanded after HCMV reactivation in haploidentical HSCT

**DOI:** 10.1172/jci.insight.146973

**Published:** 2021-06-22

**Authors:** Elisa Zaghi, Michela Calvi, Simone Puccio, Gianmarco Spata, Sara Terzoli, Clelia Peano, Alessandra Roberto, Federica De Paoli, Jasper J.P. van Beek, Jacopo Mariotti, Chiara De Philippis, Barbara Sarina, Rossana Mineri, Stefania Bramanti, Armando Santoro, Vu Thuy Khanh Le-Trilling, Mirko Trilling, Emanuela Marcenaro, Luca Castagna, Clara Di Vito, Enrico Lugli, Domenico Mavilio

**Affiliations:** 1 Unit of Clinical and Experimental Immunology, IRCCS Humanitas Research Hospital, Rozzano, Milan, Italy.; 2 BIOMETRA, Università degli Studi di Milano, Milan, Italy.; 3 Laboratory of Translational Immunology,; 4 Institute of Genetic and Biomedical Research, UoS Milan, National Research Council, and Genomic Unit,; 5 Bone Marrow Transplant Unit, and; 6 Molecular Biology Section, Clinical Investigation Laboratory, IRCCS Humanitas Research Hospital, Milan, Italy.; 7 Institute for Virology, University Hospital Essen, University Duisburg-Essen, Essen, Germany.; 8 Department of Experimental Medicine, University of Genoa, Genoa, Italy.; 9 Flow Cytometry Core, IRCCS Humanitas Research Hospital, Milan, Italy.

**Keywords:** Hematology, Immunology, Bone marrow transplantation, Innate immunity, NK cells

## Abstract

Haploidentical hematopoietic stem cell transplantation (h-HSCT) represents an efficient curative approach for patients affected by hematologic malignancies in which the reduced intensity conditioning induces a state of immunologic tolerance between donor and recipient. However, opportunistic viral infections greatly affect h-HSCT clinical outcomes. NK cells are the first lymphocytes that recover after transplant and provide a prompt defense against human cytomegalovirus (HCMV) infection/reactivation. By undertaking a longitudinal single-cell computational profiling of multiparametric flow cytometry, we show that HCMV accelerates NK cell immune reconstitution together with the expansion of CD158b1b2j^pos^/NKG2A^neg^/NKG2C^pos^/NKp30^lo^ NK cells. The frequency of this subset correlates with HCMV viremia, further increases in recipients experiencing multiple episodes of viral reactivations, and persists for months after the infection. The transcriptional profile of FACS-sorted CD158b1b2j^pos^ NK cells confirmed the ability of HCMV to deregulate NKG2C, NKG2A, and NKp30 gene expression, thus inducing the expansion of NK cells with adaptive traits. These NK cells are characterized by the downmodulation of several gene pathways associated with cell migration, the cell cycle, and effector-functions, as well as by a state of metabolic/cellular exhaustion. This profile reflects the functional impairments of adaptive NK cells to produce IFN-γ, a phenomenon also due to the viral-induced expression of lymphocyte-activation gene 3 (LAG-3) and programmed cell death protein 1 (PD-1) checkpoint inhibitors.

## Graphical abstract

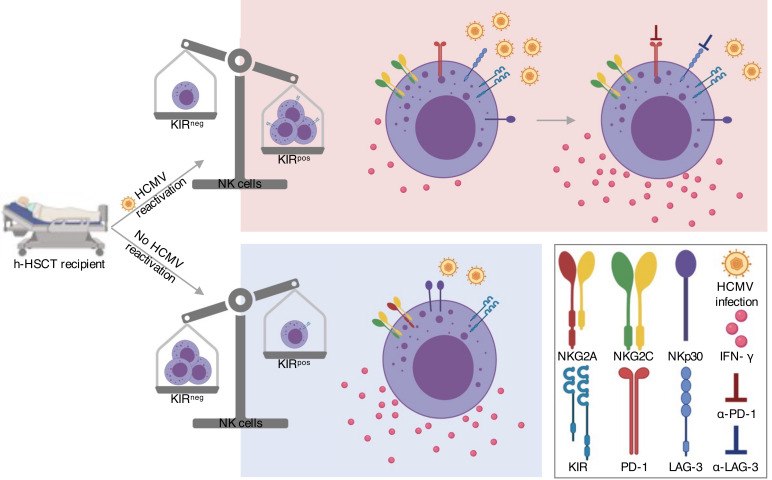

## Introduction

Haploidentical hematopoietic stem cell transplantation (h-HSCT) is a life-saving therapeutic strategy to cure a wide range of hematologic malignancies (1, 2). In particular, myeloablative (MA) and non-MA T cell replete h-HSCT protocols with posttransplant cyclophosphamide (PT-Cy) administration showed remarkably positive clinical outcomes in terms of overall survival and progression-free survival. However, the clinical benefits of h-HSCT are still hampered by life-threatening side effects including the occurrence of opportunistic viral infections as a consequence of the prolonged immune-deficiency after transplantation. In this scenario, acute and chronic human cytomegalovirus (HCMV) replication, either originating from a primary infection or from the reactivation of a preexistent latent virus in the graft or in the recipient, represents the most frequent adverse clinical condition, occurring in 35%–50% of patients who have undergone h-HSCT (3–8).

Natural Killer (NK) cells are the first donor-derived lymphocytes that immune-reconstitute after h-HSCT, thus providing a prompt immune-surveillance against invading pathogens and tumor cells surviving despite conditioning regimens (9, 10). Circulating NK cell subsets are defined by the surface expression of CD56 and CD16. CD56^bright^/CD16^neg^ (CD56^br^) cells represent a regulatory subset accounting for about 10% of NK lymphocytes in peripheral blood (PB) able to secrete inflammatory cytokines and to regulate immune cross-talks. Terminally-differentiated CD56^dim^/CD16^pos^ (CD56^dim^) NK cells represent the majority of these innate lymphocytes in PB exerting cytotoxic functions (11–13). In addition to these two conventional subsets, other NK cell subpopulations arise in response to pathologic stimuli. In this regard, HCMV replication promotes the expansion of CD56^neg^/CD16^pos^ (CD56^neg^) NK cells that are otherwise almost undetectable under homeostatic conditions (6, 14, 15). CD56^neg^ NK cells were first reported as a highly impaired subset expanded in chronic HIV-1 and HCV infections (15–18). Other studies later demonstrated that viruses greatly impact NK cell homeostasis and maturation by inducing the expansion of NK cells exhibiting adaptive traits and showing the strongest effector-functions (i.e., higher production of IFN-γ) when reencountering the same pathogen (19–22). These adaptive NK cells have been extensively characterized in mice, where the binding of the Ly49H receptor to the murine cytomegalovirus glycoprotein m157 activates the NK cells to clear the virus (23). Unlike murine models, the precise receptor(s) binding HCMV antigens and the markers characterizing adaptive NK cells in humans are still being debated. Currently, human adaptive NK lymphocytes are defined as terminally-differentiated NKG2C^pos^ cells lacking the expression of the inhibitory receptor NKG2A and expressing high levels of CD57 and killer immunoglobulin-like receptors (KIRs) (11, 24). However, the appearance of adaptive NK cells does not necessary require the engagement of NKG2C, although this activating NK receptor (aNKR) exhibits specificity for the HCMV-encoded UL40 protein. Accordingly, the expansion of HCMV-induced KIR^pos^ NK cells with adaptive traits has been also found in NKG2C-deficient subjects and in patients receiving cord blood grafts from NKG2C^–/–^ donors, suggesting that NKG2C is a marker rather than an essential mediator of this process. Hence, these findings indicate the presence of alternative NKRs and pathways involved in the generation of adaptive NK cells (25, 26). In this regard, adaptive NK cells share with memory T cells an epigenetic reprogramming with genome-wide DNA methylation patterns responsible for higher IFN-γ production in response to HCMV infection (27–29). Moreover, and similar to memory T cells, adaptive NK cells can exhibit dysfunctional features, exhausted phenotypes, and increased levels of checkpoint inhibitors PD-1 and LAG-3 following chronic stimulation (24, 30–33).

In a longitudinal setting, the present study characterizes the kinetics as well as the cellular and molecular features of NK cell subsets in response to in vivo HCMV infection/reactivation in recipients who underwent h-HSCT as therapy for hematologic malignancies.

## Results

### Impact of HCMV infection/reactivation on the distribution and phenotype of immune-reconstituting NK cell subsets.

**Figure 1:**
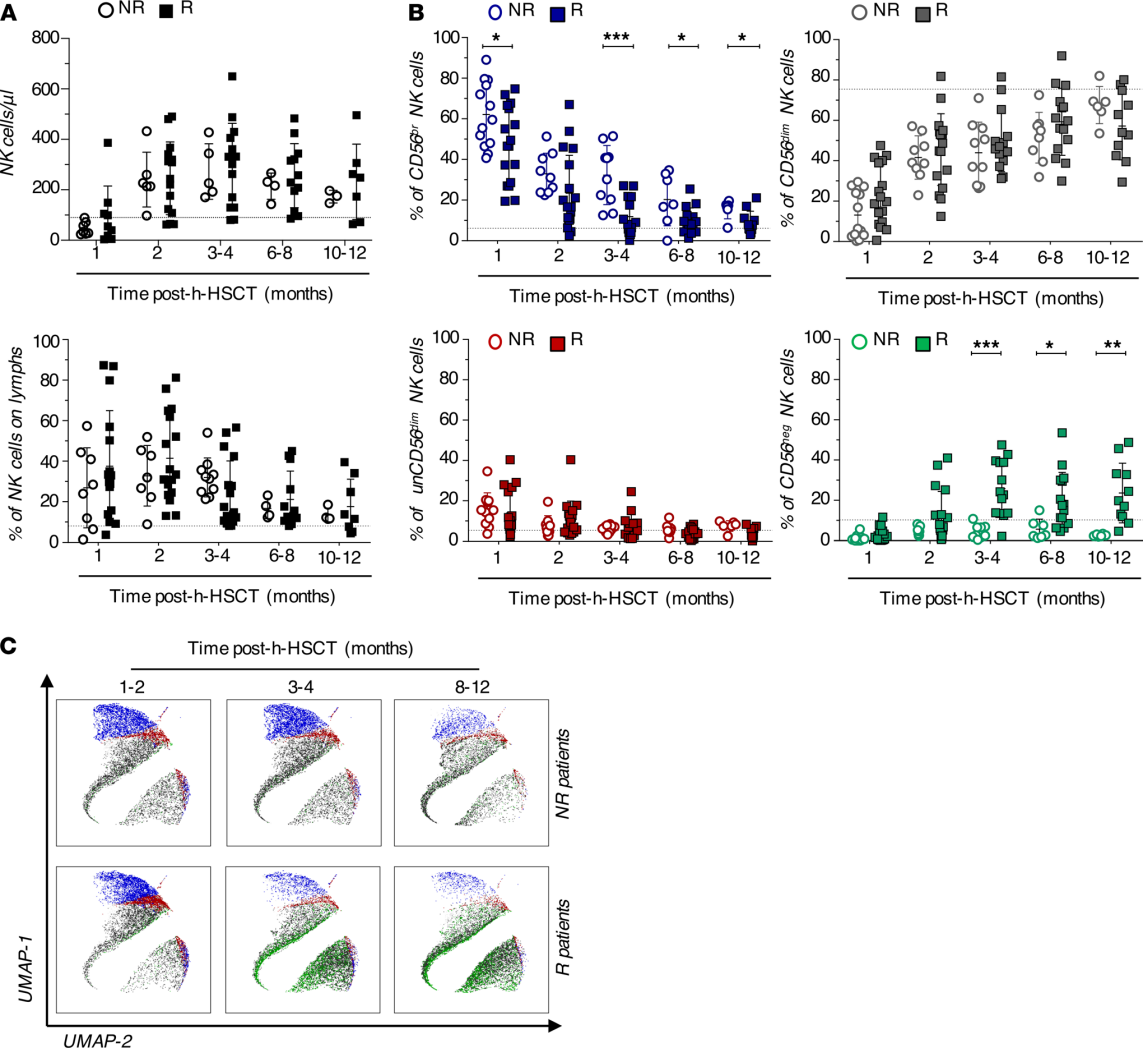
Impact of HCMV infection/reactivation on the kinetics of NK cell subset immune-reconstitution in patients who underwent h-HSCT. (**A** and **B**) Summary statistical graphs showing (**A**) the absolute counts (cells/μL; mean ± SD) of circulating NK cells (left) and the frequencies (%; mean ± SD) of total NK cells (right) and (**B**) of CD56^br^ (blue), CD56^dim^ (black), unCD56^dim^ (red), and CD56^neg^ (green) NK cell subsets in the PB of recipients either experiencing (R, *n =* 18) HCMV reactivation/infection or not (NR, *n =* 14) at different time points after h-HSCT. Dashed lines represent mean values of each variable in the related HDs. Unpaired *t* test, NR versus R recipients. *P < 0.05, **P < 0.01, ***P < 0.001. (**C**) UMAP plots showing the distribution of NK cell subsets after h-HSCT. CD56^br^ (blue), unCD56^dim^ (red), CD56^dim^ (black), and CD56^neg^ (green) NK cells in NR (*n =* 10; upper panels) and in R (*n =* 18; lower panels) recipients are followed longitudinally at three different time points (1–2, 3–4, and 8–12 months) after h-HSCT. NK cell subsets are overlaid with the total NK cell distribution (gray background).

To investigate the impact of HCMV infection/reactivation on NK cell immune-reconstitution (IR) after h-HSCT, we longitudinally characterized the phenotype of viable CD14^neg^/CD3^neg^/Lineage^neg^ lymphocytes within peripheral blood mononuclear cells (PBMCs) from both h-HSCT recipients and their relative HSC donors (Supplemental Figure 1; supplemental material available online with this article; https://doi.org/10.1172/jci.insight.146973DS1). To this end, h-HSCT recipients were stratified into two groups either experiencing (R) or not (NR) HCMV infection/reactivation, generally during the first two months after the transplant (Table 1 and ref. 3). We first observed that the absolute counts and frequencies of total NK cells did not significantly differ between R and NR recipients at any of the time-points analyzed (Figure 1A). We and others have previously reported that donor-derived immune-reconstituting CD56^br^ and CD56^dim^/CD16^neg^ (unCD56^dim^) NK cell subsets expand soon after h-HSCT and outnumber terminally-differentiated CD56^dim^ NK cells (10, 34). We showed that HCMV infection/reactivation accelerated the maturation of NK cells by significantly decreasing the frequencies over time of CD56^br^ NK cells in R compared with NR. This phenomenon was counterbalanced by the HCMV-induced expansion, soon after HCMV infection/reactivation, of CD56^neg^ NK cells, a subset that is very low or undetectable in NR recipients (Figure 1B).

**Figure 2:**
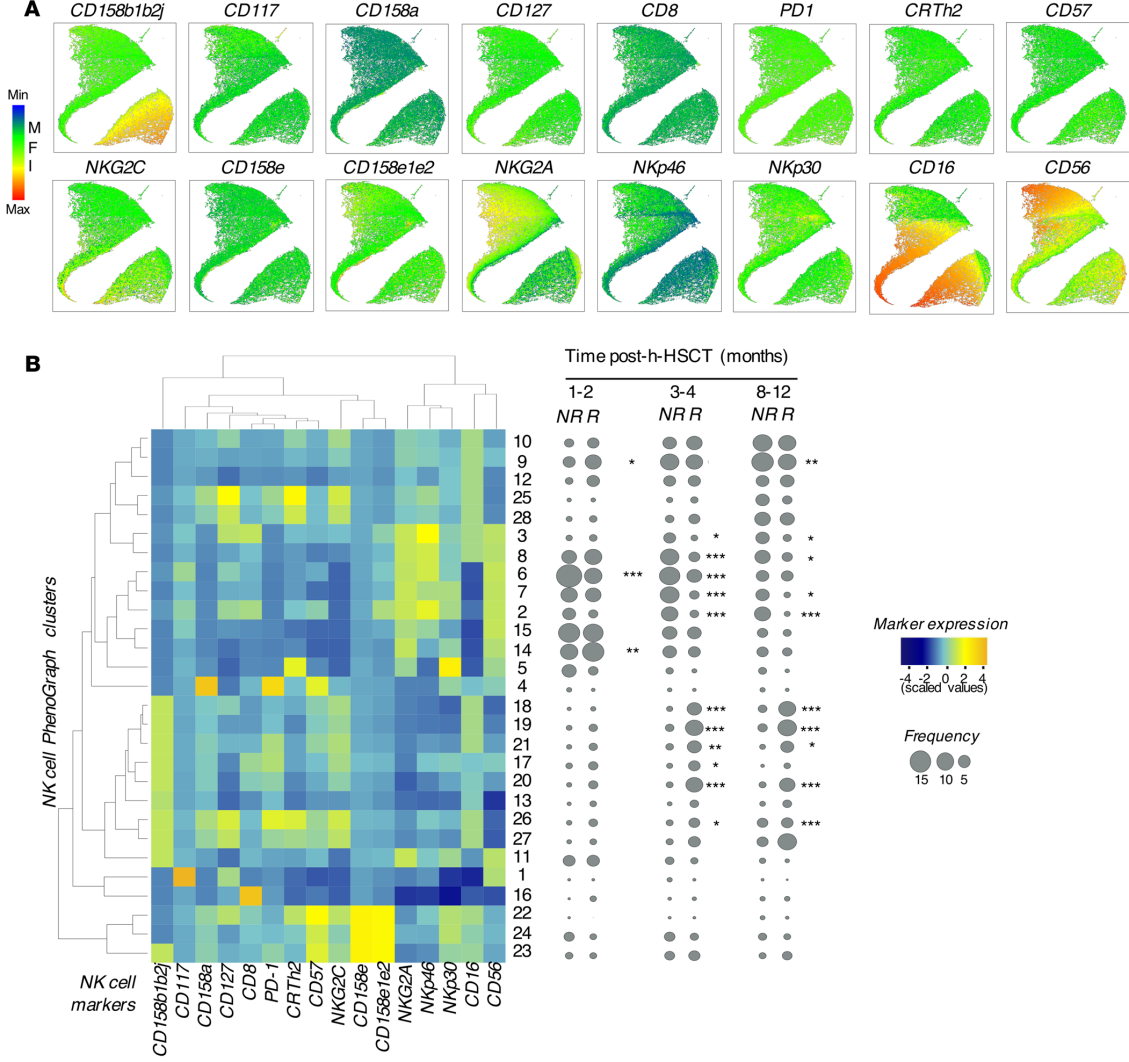
High-dimensional single-cell analysis of NK cell phenotype in h-HSCT recipients. (**A**) UMAP plots showing the mean fluorescence intensity (MFI) of markers on concatenated NK cells (2,000 cells/sample) from both NR and R recipients at different time points after h-HSCT. (**B**) Heatmap showing the expression as a percentage of NK cell markers (columns) on the 28 clusters (rows) assessed by PhenoGraph from h-HSCT recipients regardless of HCMV reactivation/infection (left). Statistical balloon plots showing the median frequencies of each PhenoGraph NK cell cluster in R (*n =* 13) and NR (*n =* 6) recipients at three different time points (1–2, 3–4, and 8–12 months) after h-HSCT (right). Multiple *t* tests, NR versus R recipients. *P < 0.05, **P < 0.01, ***P < 0.001.

To gain greater insight into the NK cell phenotypic features of NK cells associated with HCMV reactivation/infection, flow cytometry data were concatenated and analyzed with the PhenoGraph unsupervised clustering algorithm (35). The results obtained were visualized by using Uniform Manifold Approximation and Projection (UMAP) to simplify the visualization of marker expression at the single-cell level (Figure 2A).

This approach first confirmed that HCMV infection/reactivation modified the kinetics of NK cell subset IR and induced the expansion of CD56^neg^ NK cells, whose high frequencies persist for at least 1 year after h-HSCT (Figure 1, B and C).

**Figure 3:**
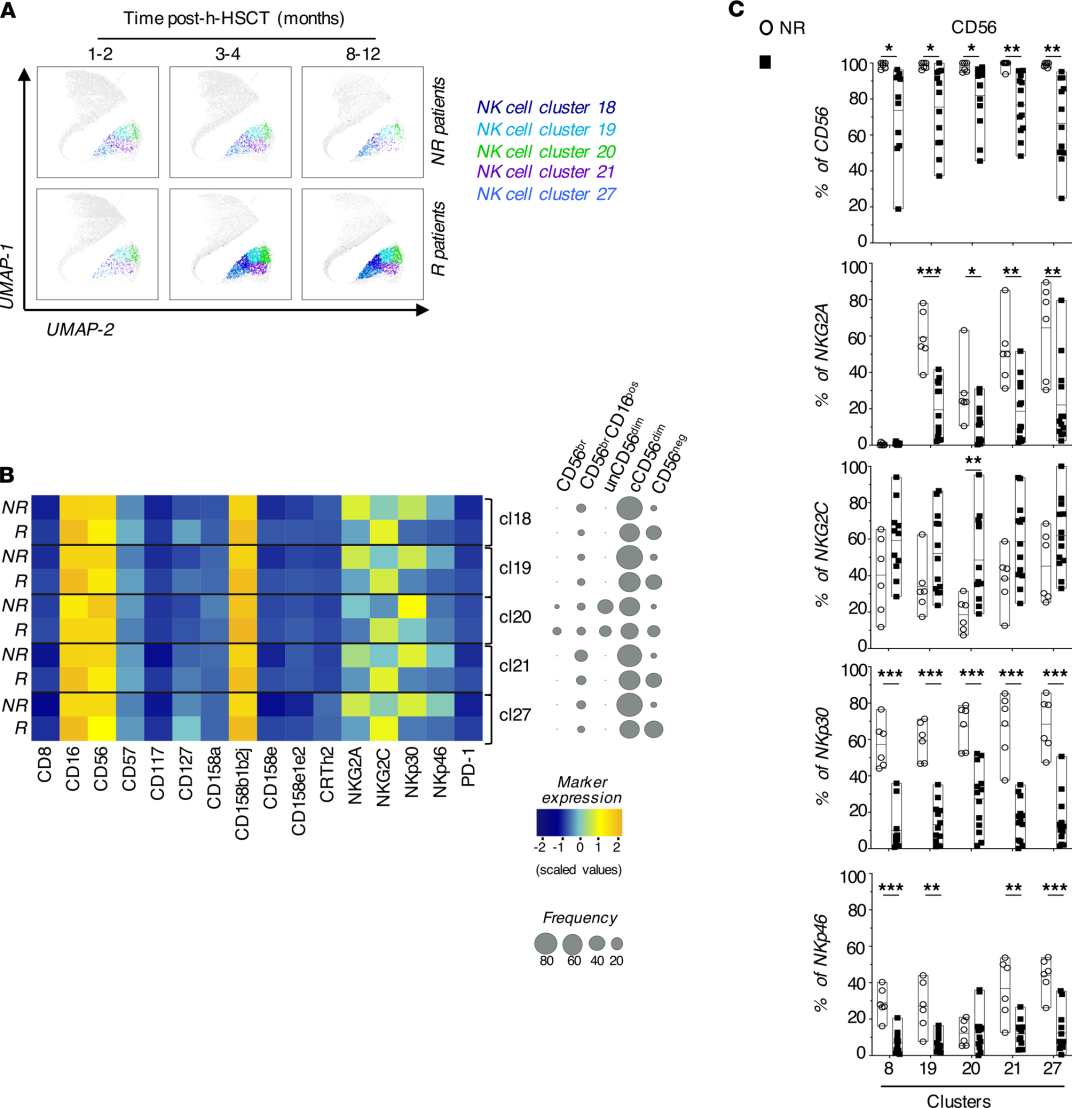
HCMV shapes the NK cell receptor repertoire in patients who underwent h-HSCT. (**A**) UMAP plots depicting the distribution of 18, 19, 20, 21, and 27 clusters in NR (upper panels) and R (lower panels) recipients before (1–2 months), soon (3–4 months), and late (8–12 months) after HCMV infection/reactivation. NK cell clusters are overlaid with the total NK cell distribution (gray background). (**B**) Heatmap showing the expression of NK cell markers and receptors on clusters 18, 19, 20, 21, and 27 in NR (*n =* 6) and in R (*n =* 13) h-HSCT recipients (left). Balloon plots showing the distribution of NK cell subsets in each of the 5 clusters in NR and R recipients at 8–12 months after h-HSCT (right). (**C**) Summary statistical graphs reporting the frequencies (%) of NK cell markers differentially expressed in clusters 18, 19, 20, 21, and 27 in NR (*n =* 6) and R (*n =* 13) recipients at 8–12 months after h-HSCT. Unpaired *t* test. *P < 0.05, **P < 0.01, ***P < 0.001.

Through PhenoGraph analysis, we also identified 28 phenotypically distinct clusters of NK cells that were followed longitudinally before (1–2 months), soon after (3–4 months) and later after (8–12 months) the onset of this opportunistic viral infection. While the overall frequencies of the 28 NK cell clusters were similar between R and NR before HCMV infection/reactivation, the frequencies of the 5 distinct NK cell clusters (18, 19, 20, 21, 27) significantly increased early after the onset of this opportunistic infection at 3 to 4 months following h-HSCT and remained high even 1 year after the transplant (Figure 2B and Figure 3A). These 5 clusters were mainly enriched within mature CD56^dim^ NK cells at all time points analyzed and expressed high levels of CD158b1b2j (KIR2DL2/2DL3/2DS2) and CD16, two surface markers of “licensed” human NK cells (11). Interestingly, the HCMV infection modified the phenotypic repertoires of mature NK cells belonging to the above-mentioned clusters by inducing the expression of NKG2C and by decreasing the surface levels of NKG2A, NKp30 and NKp46. The differential modulation of these NKRs have been associated with adaptive NK cells together with the increased expression of CD57 (11). Surprisingly, our data did not show any difference between R and NR in the surface levels of CD57 on NK cells comprised within these 5 clusters (Figure 3, B and C). Of note, our PhenoGraph analyses also showed that the expression of CD56 is decreased in R compared with NR. This phenomenon was due to the expansion of CD56^neg^ NK cells restricted to R and confirms our results obtained with manual gating and UMAP (Figure 3C and Figure 1, B and C).

### HCMV drives the expansion of long-lasting CD158b1b2j^pos^/NKG2A^neg^/NKG2C^pos^/NKp30^lo^ NK cells.

**Figure 4:**
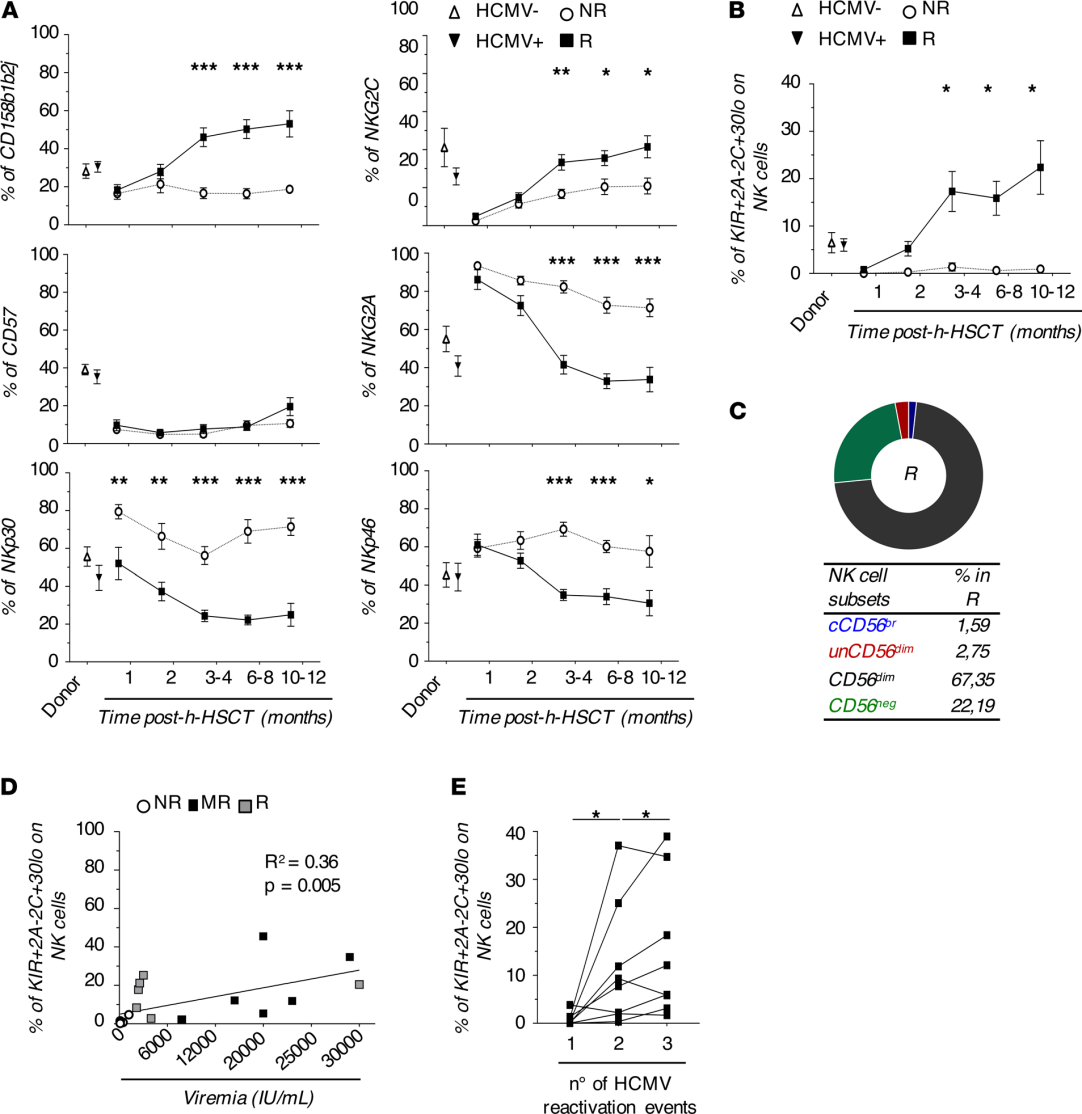
Expansion of long-lasting CD158b1b2j^pos^/NKG2A^neg^/NKG2C^pos^/NKp30^lo^ NK cell in response to HCMV infection/reactivation. (**A**) Summary statistical graphs showing the frequency (%; mean ± SEM) of NK cell receptors differentially expressed in NK cell clusters 18, 19, 20, 21, and 27 in HCMV- (*n =* 6) and HCMV+ (*n =* 11) donor PBMCs and in NR (*n =* 14) and in R (*n =* 18) patients who underwent h-HSCT at different time points after the transplant. Unpaired *t* test. (**B**) Summary statistical graph showing the frequency (%, mean ± SEM) of CD158b1b2j^pos^/NKG2A^neg^/NKG2C^pos^/NKp30^lo^ NK cells on total NK cells in HCMV- (*n =* 6) and HCMV+ (*n =* 11) donor PBMCs and NR (*n =* 14) and R (*n =* 18) recipients at different time points after h-HSCT. (**C**) Pie chart showing the relative frequencies of NK cell subsets within the CD158b1b2j^pos^NKG2A^neg^NKG2C^pos^NKp30^lo^ NK cell population of R (*n =* 18) recipients at 8–12 months after h-HSCT. (**D**) Summary statistical graph showing the correlation between the frequency (%) of CD158b1b2j^pos^/NKG2A^neg^/NKG2C^pos^/NKp30^lo^ NK subset on total NK cells and HCMV pick of viremia (IU/mL) in R either experiencing single (R, *n =* 5) or multiple (MR, *n =* 6) HCMV reactivations at 8–12 months after h-HSCT. Data from NR patients (*n =* 8) at 8–12 months after h-HSCT are inserted as negative control. (**E**) Summary statistical graphs showing the frequency (%) of CD158b1b2j^pos^/NKG2A^neg^/NKG2C^pos^/NKp30^lo^ on total NK cells in MR patients (*n =* 7) experiencing 3 longitudinal reactivation events (n° = 1, 2, and 3). Blood samples of MR were analyzed at the first available time point (range: 0–21 days) after the pick of viremia defining HCMV reactivation events. Paired *t* test. *P < 0.05, **P < 0.01, ***P < 0.001.

After the identification of those NKRs differentially expressed on NK cells in response to HCMV infection/reactivation, we evaluated the kinetics of these receptors on total immune-reconstituting NK cells. Our results showed a statistically significant increase of CD158b1b2j and NKG2C together with a statistically significant decrease of NKp30, NKp46, and NKG2A starting from the third month after h-HSCT and soon after the onset of this opportunistic viral infection. Again, we did not observe any changes in the CD57 surface expression in R compared with NR. Interestingly, our data demonstrated that the amounts of NKp30 were significantly lower in R compared with NR even prior to clinically-recognized HCMV replication, and the degree of differential expression on NK cells between these two groups of recipients further increased following the onset of this opportunistic viral infection (Figure 4A).

We then combined the kinetics of the above-mentioned NKRs in order to possibly identify a specific NK cell subset highly responsive to HCMV infection/reactivation. Although both NKp30 and NKp46 were significantly decreased on immune-reconstituting NK cells early after HCMV infection\reactivation, we focused our analysis only on NKp30 since this Natural Cytotoxic Receptor (NCR) showed the highest degree of differential expression between R and NR at 8–12 months after h-HSCT (Figure 3, B and C). The manual gating on CD158b1b2j^pos^/NKG2A^neg^/NKG2C^pos^/NKp30^lo^ cells identified a potentially novel NK cell subset that arose soon after HCMV infection/reactivation, starting from the third month after h-HSCT and persisting at significantly higher levels in R compared with NR until 1 year after the transplant (Figure 4B). As expected, CD158b1b2j^pos^/NKG2A^neg^/NKG2C^pos^/NKp30^lo^ NK cells were mostly comprised within the subset of mature CD56^dim^ and CD56^neg^ NK cells (Figure 4C).

We then evaluated the clinical impact of HCMV viremia in the expansion of this NK cell subset, also taking into account that 9 out of 21 R recipients experienced multiple viral reactivation episodes (MR; Table 1). Our data showed that the frequency of CD158b1b2j^pos^/NKG2A^neg^/NKG2C^pos^/NKp30^lo^ NK cells positively correlated with HCMV viremia and was higher in MR compared with other R recipients (Figure 4D). Moreover, the percentages of this NK cell subset increased even more after every new HCMV reactivation event in MR, suggesting that these cells might remember and rapidly recall the previous viral challenges through a phenomenon of trained immunity (Figure 4E).

Of note, each one of the 4 NKRs that characterizes this NK cell subset showed different kinetics during MR of HCMV. While the expressions of CD158b1b2j and NKG2C increased after each infection episode, surface levels of NKG2A and NKp30 significantly decreased after the second and third cycle of infection (Supplemental Figure 2).

### CD158b1b2j^pos^ NK cells in R patients show an adaptive transcriptional profile.

To assess if our newly disclosed NK cell subset was endowed with adaptive traits, we investigated its transcriptional profile by performing RNA-Seq. We FACS-sorted NK cells based on their expression of CD158b1b2j from recipients at 7–12 months after h-HSCT. We adopted this strategy to obtain a sufficient number of highly pure NK cells expressing CD158b1b2j that should identify those NK cell clusters highly enriched in R compared with NR (Figure 3B and Figure 4A).

As expected, the analysis of NK cell subset distribution on FACS-sorted CD158b1b2j^pos^ (KIR^pos^) and CD158b1b2j^neg^ (KIR^neg^) NK cells demonstrated that KIR^pos^ NK cells from R and NR had a lower frequency of CD56^br^ and unCD56^dim^ NK cells in comparison to KIR^neg^ NK cells from R. Moreover, CD56^neg^ NK cells were mainly present among NK cells from R irrespective of the KIR^pos^ and KIR^neg^ status (Supplemental Figure 3).

**Figure 5:**
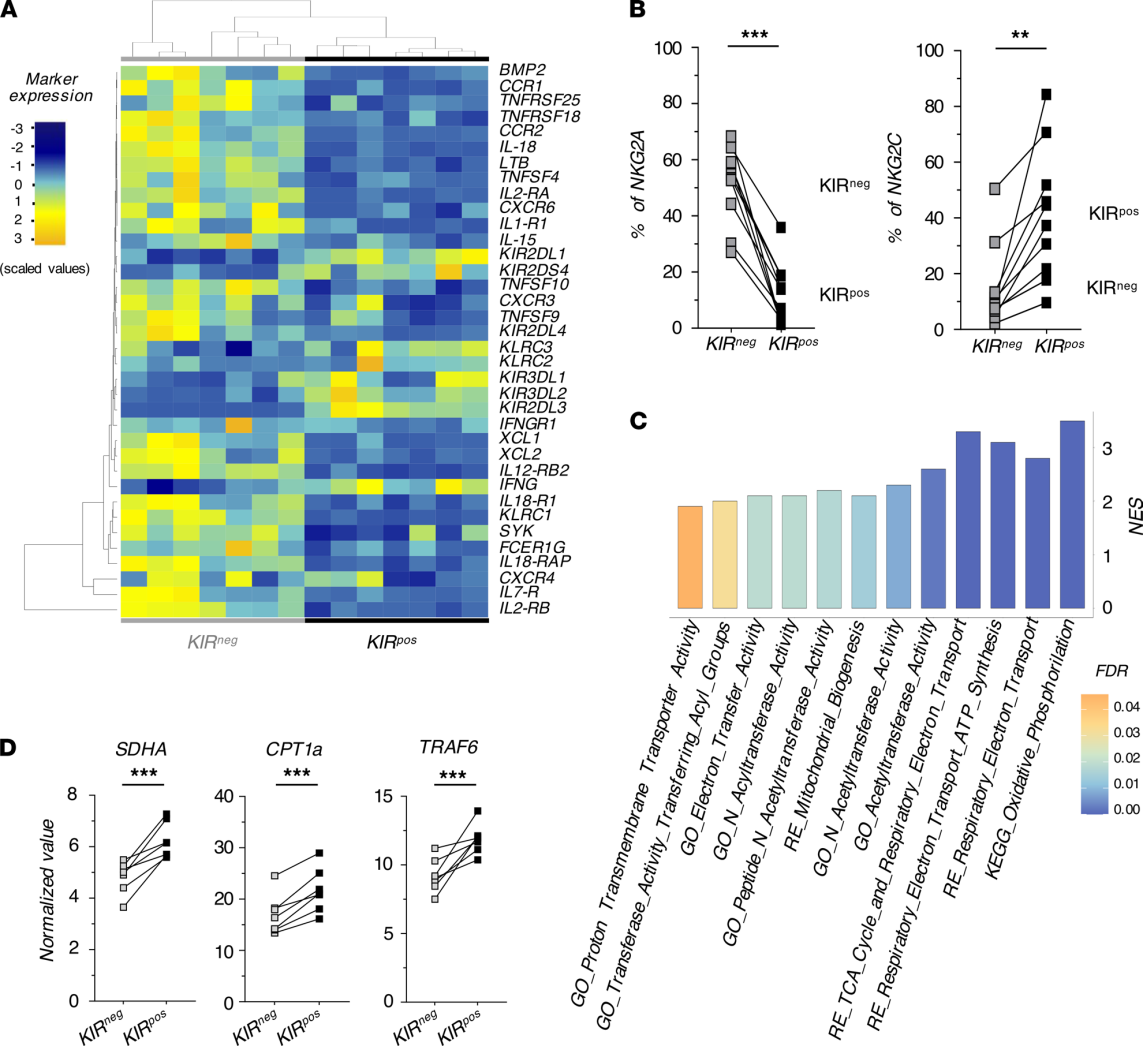
KIR^pos^ NK cells in patients who underwent R h-HSCT have an adaptive transcriptional profile. (**A**) Heatmap showing the significantly deregulated genes governing the NK cell functions in KIR^neg^ (gray) compared with KIR^pos^ (black) NK cells from R. (**B**) Summary statistical graphs showing the expression (%; mean ± SD) of NKG2A (left) and NKG2C (right) in KIR^neg^ and KIR^pos^ NK cells from R at 10–12 months after h-HSCT. Dashed lines represent the mean values in relative HDs. Paired *t* test. (**C**) Gene sets involved in mitochondrial respiration and enriched in KIR^pos^ NK cells from R as retrieved from GO, KEGG, and REACTOME (RE) databases. The color of the bars denotes the FDR value. (**D**) Summary statistical graphs showing the normalized value (x10^2^) of *SDHA*, *CPT1a*, and *TRAF6* genes in KIR^neg^ and KIR^pos^ of FACS-sorted NK cells from R. *P* value adjusted. *P < 0.05, **P < 0.01, ***P < 0.001.

The Principal Component Analysis (PCA) first revealed that KIR^pos^ NK cells clustered separately from their KIR^neg^ counterparts in R, thus indicating the existence of different gene expression profiles between them. Accordingly, 2,684 differentially expressed genes (DEGs) separated the two comparison groups (Supplemental Figure 4, A and B). Among them, KIR^pos^ NK cells expressed lower levels of *KLRC1/*NKG2A and higher levels of *KLRC2/*NKG2C compared with KIR^neg^ NK cells, thus confirming our flow cytometry data (Figure 5, A and B). These RNA-Seq data also highlighted the upregulation in KIR^pos^ NK cells of *KLRC3*/NKG2E and other KIR genes including *KIR2DL1, KIR3DL1, KIR3DL2*, and *KIR2DS4*.

Moreover, and in line with previously reported epigenetic reprogramming of adaptive NK cells (27), we observed a downregulation of *FCER1G*/FcɛRγ and *SYK*/SYK in KIR^pos^ NK cells. The enrichment of *cytokines-cytokine receptor’ interaction* in KIR^neg^ NK cells (KEGG; Normalized enrichment score [NES] = 3.64; False Discovery Rate [FDR] = 0.00) resembled previous experimental evidence showing a cytokine receptor imbalance in HCMV-seropositive subjects (36, 37). Indeed, *IL2RA/B*, *IL18RAP, IL18R1*, and *IL12RB2* were significantly downregulated in KIR^pos^ NK cells, indicating a general impairment of interleukin-driven responses (Figure 5A).

We then focused our transcriptional analyses on NK cell metabolism given the recently reported key role of glucose-driven glycolysis and oxidative phosphorylation (*OxPhos*) in regulating NK cell cytotoxicity and generation of adaptive NK cells (38). Our results showed that KIR^pos^ NK cells were enriched in pathways associated with mitochondrial respiration and ATP synthesis (Figure 5C). Since *OxPhos* was the first enriched pathway of KIR^pos^ NK cells within the HALLMARK database (NES = 3.8; FDR = 0.00), we analyzed the transcript levels of those genes included in this pathway. *Succinate Dehydrogenase Complex Flavoprotein Subunit A* (*SDHA*), involved in energy conversion and electron transport, was significantly upregulated in KIR^pos^ NK cells. Moreover, similarly to long-living memory T cells, adaptive NK cells showed a preference for fatty acid oxidation (FAO) to fuel *OxPhos* (39, 40). Indeed, *CPT1A* and *TRAF6*, which play a critical role in the development and the maintenance of the memory T population, were more highly expressed in KIR^pos^ compared with KIR^neg^ NK cells (Figure 5D). Overall, this transcriptional profile indicates that KIR^pos^ NK cells expanded in R are endowed with adaptive traits.

### KIR^pos^ NK cells from R patients show adaptive features upon restimulation.

**Figure 6:**
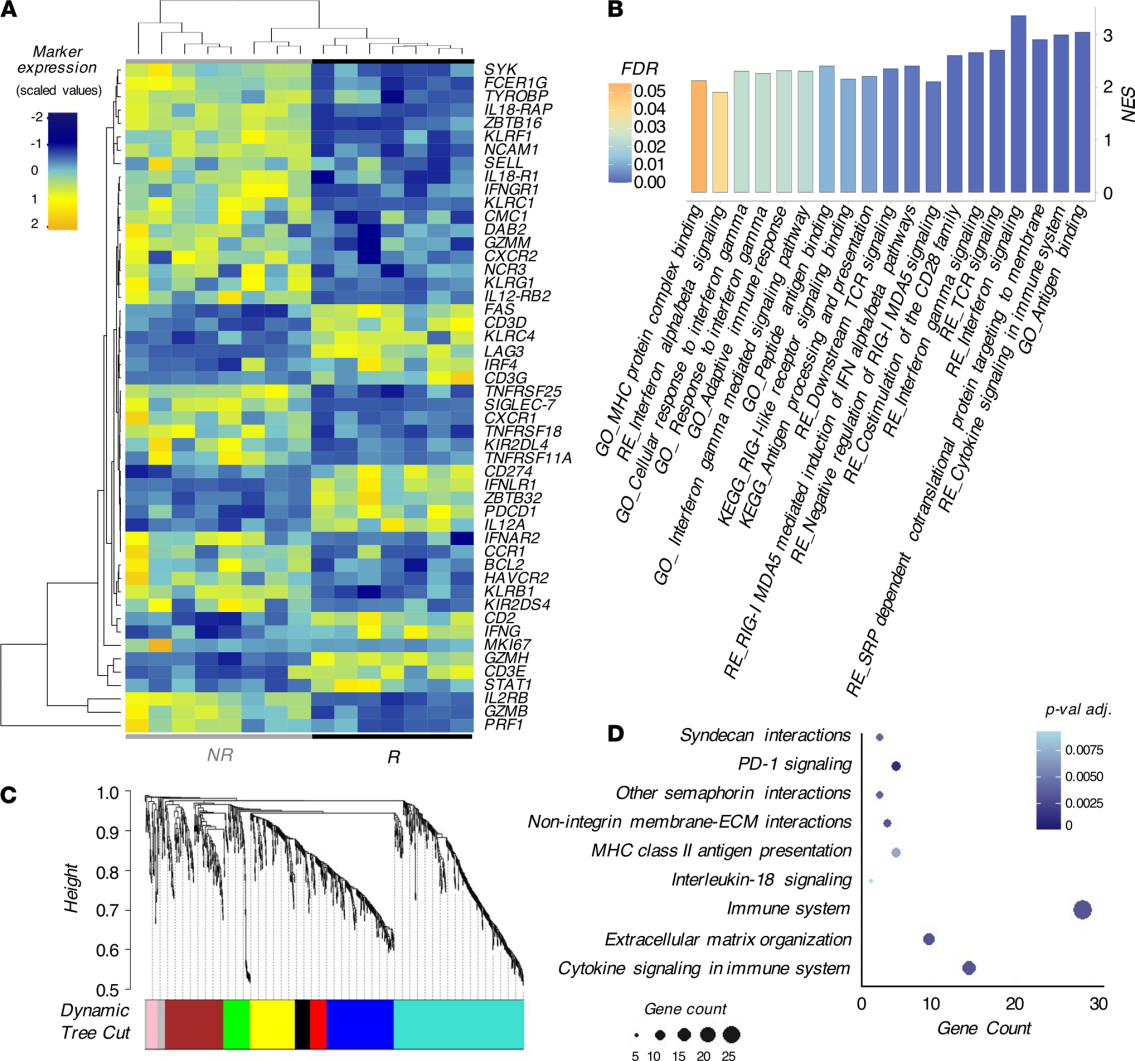
KIR^pos^ NK cells show adaptive responses following HCMV infection/reactivation. (**A**) Heatmap showing the genes significantly deregulated associated with NK cell-mediated functions in KIR^pos^ NK cells from NR versus R. (**B**) Gene sets involved in adaptive immune responses and enriched in KIR^pos^ NK cells from R retrieved from GO, KEGG, and REACTOME (RE) databases. The colors of the bar indicate the FDR value. (**C**) Cluster dendrogram and modules from WGCNA. The branches correspond to modules of highly interconnected groups of genes. Colors in the horizontal bar represent the different modules of gene expression. (**D**) Advanced bubble chart showing REACTOME pathways enriched in blue module of WGCNA. Size and color of the bubbles represent the amount of DEGs enriched in the pathway and their statistics with *P* values adjusted (*P*-adj.), respectively.

We then compared the transcriptional profiles of KIR^pos^ NK cells between NR and R. The PCA results showed that KIR^pos^ NK cells cluster differently in NR compared with R. Accordingly, 946 genes were significantly deregulated in the two groups, confirming that HCMV infection/reactivation affects the transcriptional profiles of NK cells (Supplemental Figure 4, C and D). The majority of DEGs belonged to *cytokines-cytokine receptor interaction pathways* and were enriched in NR (KEGG; NES = 2.08; FDR = 0.04). Again, we found a significant downregulation of *IL2RB*, *IL18RAP, IL18R1*, and *IL12RB2* in R compared with NR (27). Furthermore, KIR^pos^ NK cells from R downregulated several pathways relative to cell migration and genes associated with homing and chemotaxis (*CXCR1*, *CXCR2*, *CCR1*, and *CMC1*). *KLRC1/*NKG2A was one of the genes highly downregulated in R vs NR together with *NCR3/*NKp30, while *KLRC2/*NKG2C was upregulated in R versus NR (*P* value = 0.02 vs. *P* value adjusted [*P*-adj.] = 0.17). In contrast, the expression of *B3GAT1*/CD57 was similar in KIR^pos^ NK cells from NR and R (Figure 6A and Supplemental Figure 5, A and B). Taken together, these data further confirmed our flow cytometry results (Figure 3B). Our analysis also revealed a specific signature of KIR^pos^ NK cells in R characterized by higher expression of CD2 coupled with a downmodulation of CD161, NKp80, and Siglec-7, similar to other clinical and experimental settings (25, 26, 41). As expected (27), genes coded for FcɛRγ, SYK, and PLZF were downregulated in R and these result have been further confirmed by semi-quantitative real-time PCR together with EWS-activated transcript 2 (EAT-2, also called SH2D1B) downregulation. As a result of this HCMV-induced epigenetic remodeling of *IFN locus* (29), we also found that *IFNG* was significantly upregulated in R compared with NR (Figure 6A and Supplemental Figure 5C).

By using REACTOME and Gene Ontology (GO) databases, we confirmed that KIR^pos^ NK cells in R are equipped to produce high amounts of IFN-γ and share pathways with activated T lymphocytes (Figure 6B). Furthermore, the Weighted Gene Co-expression Network Analysis (WGCNA) dynamic tree cut on KIR^pos^ NK cells from NR and R generated 8 distinct gene modules in a hierarchical clustering (Figure 6C). We identified one module (depicted in blue) mostly related to features associated with of HCMV infection/reactivation. In particular, the enrichment of *signaling receptor activity* (*P*-adj. = 0.0017) in GO together with *MHC class II antigen presentation* of REACTOME (*P*-adj. = 0.0022) databases further highlighted that KIR^pos^ NK cells in R acquire traits of adaptive immunity. However, one of the main REACTOME-enriched pathways in the blue module is *PD-1 signaling*, thus indicating hallmarks of both cellular activation as well as exhaustion (Figure 6D), similar to the T cell compartment (42–44).

### Adaptive KIR^pos^ NK cells in R patients are dysfunctional.

**Figure 7:**
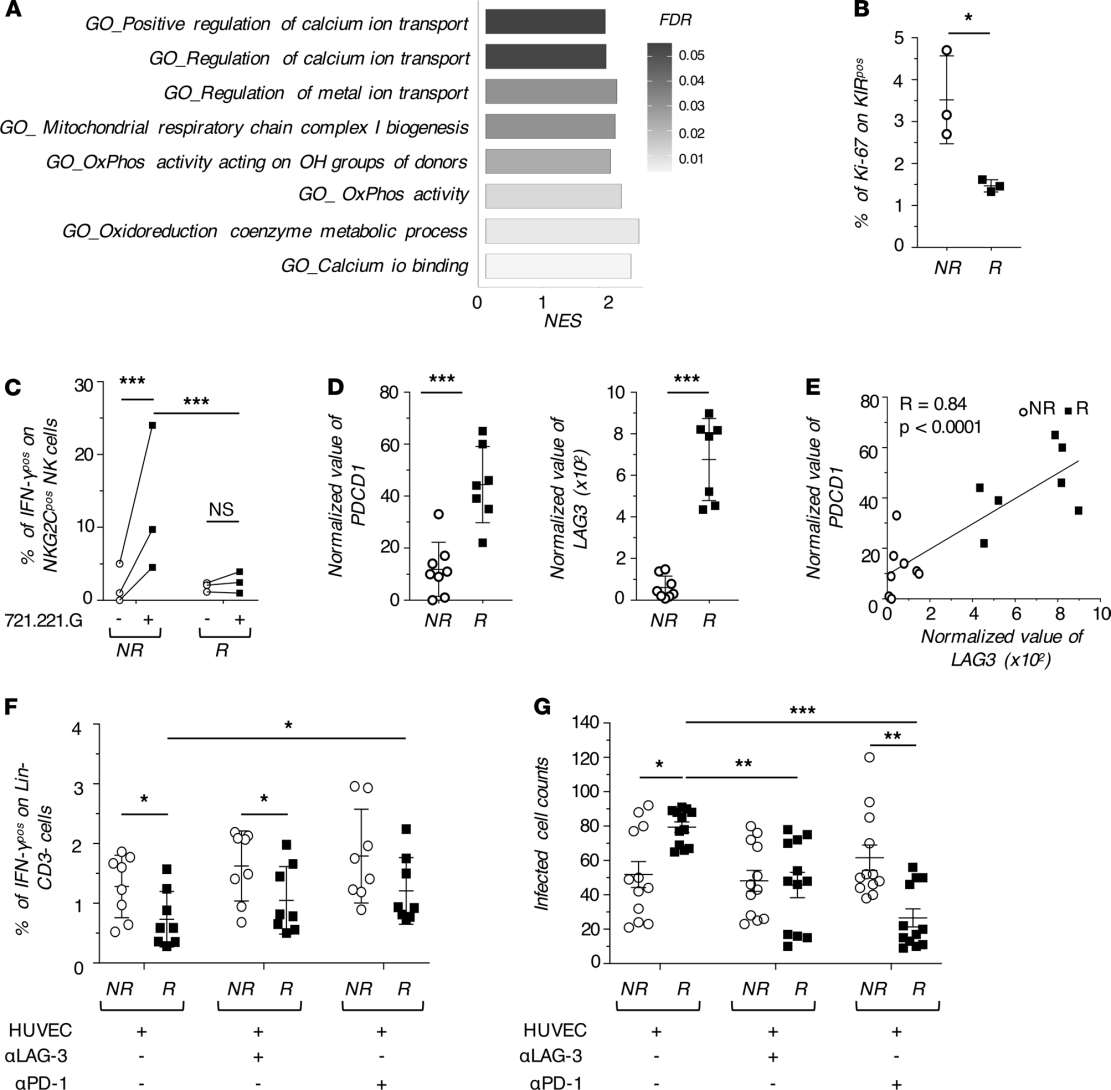
Impairment in the effector-functions of KIR^pos^ mL-NK cells. (**A**) Gene sets involved in respiratory metabolism and enriched in KIR^pos^ NK cells from NR and retrieved from GO. (**B**) Summary statistical graph showing the percentage of proliferating Ki67^pos^/KIR^pos^ NK cells (%, mean ± SD) in NR and R at 7–12 months after h-HSCT. (**C**) Summary statistical graph showing the percentage of KIR^pos^/NKG2C^pos^/IFN-γ^pos^ NK cells in NR (*n =* 3) and R (*n =* 3) recipients at 8–12 months after h-HSCT either in the absence (-) or presence (+) of 721.221.G target cell lines. One-way ANOVA with Bonferroni correction. (**D**) Summary statistical graph showing the normalized value of *PDC1* and *LAG3* gene expression in FACS-sorted KIR^pos^ NK cells from NR (*n =* 8) and R (*n =* 7). Paired *t* test. (**E**) Pearson correlation between *PDCD1* and *LAG3* gene expression on KIR^pos^ NK cells. (**F**) Summary statistical graphs showing the percentage of IFN-γ^pos^ NK cells in NR (*n =* 8) and R (*n =* 8) recipients at 8–12 months after h-HSCT, cocultured with HUVEC either in the absence (-) or presence (+) of blocking antibodies (α–PD-1 and α–LAG-3). One-way ANOVA with Bonferroni correction. (**G**) Summary statistical graphs showing the number of HUVEC cells infected with the BAC clone of the HCMV strain TB40/E expressing EGFP remained after 48 hours of coculture with KIR^pos^ NK cells from NR (*n =* 3) and R (*n =* 3) either in the absence (-) or presence (+) of blocking antibodies (α–PD-1 and α–LAG-3). One-way ANOVA with Bonferroni correction. *P < 0.05, **P < 0.01, ***P < 0.001.

Even though KIR^pos^ NK cells in R hold adaptive traits, these overtrained cells also displayed a significant reduction in pathways related to mitochondrial respiration together with an enrichment of *PD-1 signaling* (Figure 6D and Figure 7A). These features suggest that the sustained HCMV replication might be associated with an induced status of metabolic exhaustion and/or dysfunction (38). Indeed, the enrichment of *cell-cycle pathways* (REACTOME; NES = 2.05; FDR = 0.04) was restricted to KIR^pos^ in NR, while in R they were characterized by a lower expression of the proliferation marker Ki67 both at the transcriptional (log_2_FC = –1.43) and cellular levels (Figure 6A and Figure 7B). Our results further showed that KIR^pos^/NKG2C^pos^ NK cells from R produce less IFN-γ than those from NR in response to target cells expressing NKG2C ligand (Figure 7C).

In this context, the significant increase of *PDCD1*/PD-1, together with *LAG3*/LAG-3 expression in the KIR^pos^ NK cells from R compared with NR, supported our working hypothesis of additional mechanisms of cell dysfunction associated with a higher expression of inhibitory checkpoints. We found a significant positive correlation between the transcript levels of PD-1 and LAG-3 that were expressed at their highest levels in R vs NR (Figure 7, D and E). Notably, PD-1 and LAG-3 are well-known for their important role in T lymphocytes where they are upregulated upon chronic stimulation and their blockade restores cell cytotoxicity (30, 42). Therefore, we blocked either PD-1 or LAG-3 on NK cells cocultured with the HUVEC cell line expressing high levels of their ligands (Supplemental Figure 6 and ref. 45). Our results first confirmed that R-derived NK cells exhibited an impaired capacity to produce IFN-γ compared with NR-derived NK cells. The blockade of either PD-1 or LAG-3 induced a significant increase of IFN-γ production in R, which, however, did not reach the levels observed for NR (Figure 7F). We also investigated the ability of FACS-sorted KIR^pos^ NK cells from R and NR to control the HCMV infection in vitro. The results obtained demonstrated that KIR^pos^ NK cells from NR have a greater ability to control the spread of HCMV in HUVEC cells. Furthermore, and in line with our previous data, the LAG-3/PD-1 blockade restore the ability of KIR^pos^ NK cells from R to control the in vitro HCMV infection after 48 hours of coculture (Figure 7G).

These results indicate that the dysfunction/exhaustion of “adaptive” KIR^pos^ NK cells in R is mediated, at least in part, by the HCMV-induced expression of checkpoint inhibitors.

**Figure 8:**
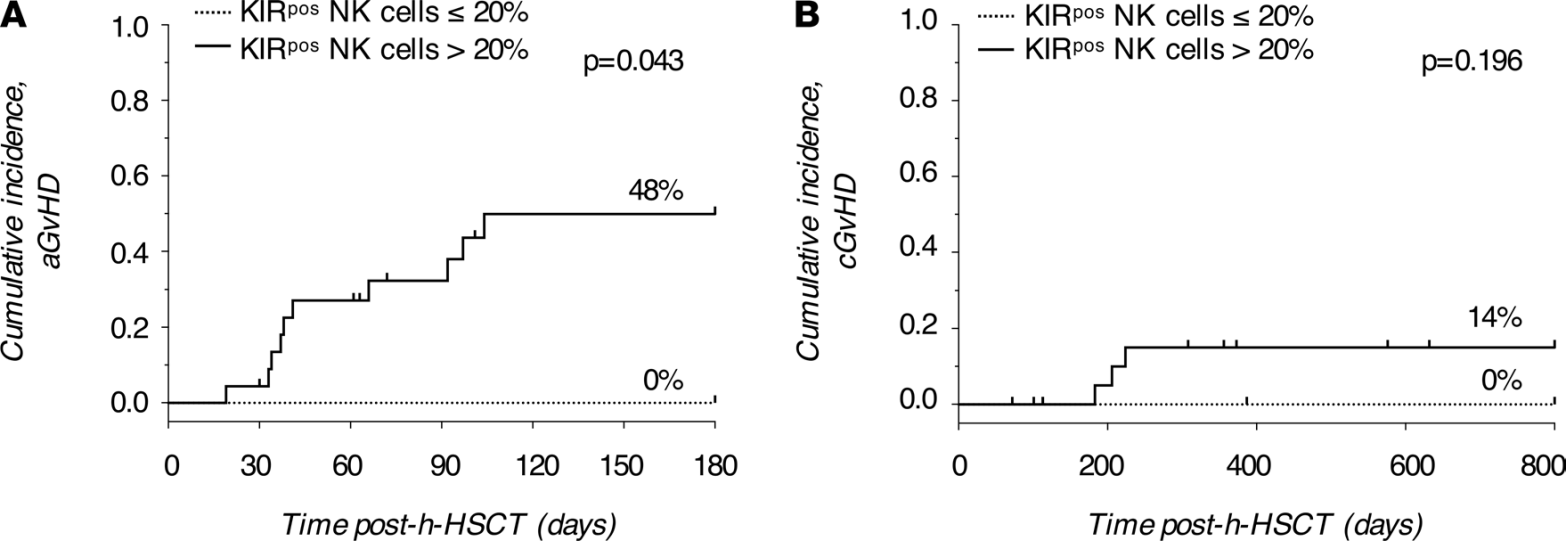
KIR^pos^ NK cell frequency correlates with the risk of GVHD. (**A**) Six months cumulative incidence of grade II-IV acute GVHD and (**B**) 2-years cumulative incidence of chronic GVHD in patients who underwent h-HSCT subdivided based on the KIR^pos^ NK cell frequencies (KIR^pos^ NK cells < 20%, *n =* 7, dotted line; KIR^pos^ NK cells > 20%, *n =* 23, solid line).

To assess the clinical relevance of our findings for h-HSCT recipients we stratified patients into two groups based on their individual frequencies of KIR^pos^ NK cells (Figure 4A). We thus set the threshold of KIR^pos^ NK cells at 20%. Intriguingly, HCMV replication, and recurrent HCMV reactivation events in particular, occurred only in patients with a frequency of KIR^pos^ NK cells higher than 20% (Table 2). We further calculate the cumulative incidence of acute and chronic graft-versus-host disease (GVHD) and the overall survival (OS). Six months cumulative incidence of grade II-IV acute GVHD was 48% versus 0% for patients whose KIR^pos^ NK cell frequency was greater than 20% and less than 20%, respectively (*P =* 0.043, Figure 8A). Consistently, the 2-years cumulative incidence of chronic GVHD was 14% when KIR^pos^ NK cells were greater than 20% versus 0% when KIR^pos^ NK cell frequency was less than 20% (*P =* 0.196; Figure 8B). The 4-years OS was 61% and 71% for patients whose KIR^pos^ NK cells were greater than 20% and less than 20%, respectively (*P =* 0.153, Supplemental Figure 7).

## Discussion

The PT-Cy h-HSCT platform allows identification of a donor for almost every patient and induces immunologic tolerance between donor and recipient (46, 47). However, due to the prolonged immunodeficiency after transplantation, the onset of opportunistic viral infections still significantly impairs the outcome of h-HSCT (2–5, 7, 8, 48). We show here that the occurrence of HCMV infection/reactivation in patients who underwent h-HSCT greatly impacts the homeostasis of NK cells by inducing the expansion of dysfunctional CD158b1b2j^pos^/NKG2A^neg^/NKG2C^pos^/NKp30^lo^ long-living lymphocytes endowed with adaptive traits and belonging to both CD56^dim^ and CD56^neg^ NK cell subsets.

The appearance of CD56^neg^ NK cells had been observed in response to several chronic viral infections *in vivo*, although the mechanisms of this phenomenon have never been fully recapitulated in vitro (15). Our longitudinal analysis from patients who underwent h-HSCT demonstrated that the occurrence of HCMV replication accelerates NK cell maturation by decreasing the frequencies of CD56^br^ NK cells and by inducing a significant expansion of CD56^neg^ NK cells. However, the onset of HCMV infection/reactivation does not affect the frequencies of immune-reconstituting CD56^dim^ NK cells, further supporting the hypothesis that CD56^neg^ NK cells likely originate from activated CD56^dim^ NK cells with which they share phenotypic similarities (17, 20, 49–51).

Our unbiased computational approach at the single-cell level on flow cytometry data revealed that HCMV infection/reactivation deregulates the surface expression of several NKRs that, combined together, identify a potentially novel CD158b1b2j^pos^/NKG2A^neg^/NKG2C^pos^/NKp30^lo^ subset of mature NK cells. This population appears soon after the onset of this viral infection and persists for at least 1 year following the transplant. Surprisingly, and different from HCMV-seropositive healthy donors (HD), NK cells isolated from R do not have higher surface levels of CD57, a maturation marker widely associated with adaptive NK cells (11, 21, 24). These experimental findings indicate that the modulation of CD57 expression cannot be used in this *in vivo* h-HSCT clinical setting to track adaptive NK cells in response to HCMV infection/reactivation. We also show that CD158b1b2j^pos^/NKG2A^neg^/NKG2C^pos^/NKp30^lo^ NK cells belong to both CD56^dim^ and CD56^neg^ NK cells, further confirming the impact of this HCMV to accelerate NK cell terminal differentiation. In this context, the loss of CD56 might identify those “adaptive” NK cells that become impaired/exhausted in response to chronic inflammatory conditions and viral infections, as previously reported (15, 17, 20).

The expansion of CD158b1b2j^pos^/NKG2A^neg^/NKG2C^pos^/NKp30^lo^ NK cells directly correlates with HCMV viral loads and their frequencies are even higher in MR patients, thus indicating that viral rechallenges greatly influence NK cell homeostasis and promote the expansion of adaptive NK cells. Therefore, high frequencies of adaptive NK cells require increased levels of viral burdens to boost their responses against HCMV in immune-compromised patients during the first weeks following h-HSCT. Given the heterogeneity of the human adaptive NK cell phenotype, we then assessed the transcriptional profile of FACS-sorted KIR^pos^ and KIR^neg^ NK cells in R, considering that KIR^pos^ NK cells are highly expanded in R even at 1 year after h-HSCT. The expression of KIRs as markers of licensed and adaptive NK cells in viral infections has been well demonstrated (11, 21). We also compared the molecular fingerprints of KIR^pos^ NK cells in R vs NR, since the latter cohort of recipients has lower but still adequate frequencies of circulating KIR^pos^ NK cell. As expected (19, 20), and in line with our flow cytometry results, KIR^pos^ NK cells in R are characterized by the downregulation of *KLRC1*/NKG2A and *NCR3/*NKp30 and the upregulation of *KLRC2/*NKG2C when compared with their KIR^neg^ counterparts. KIR^pos^ NK cells also express higher levels of other KIRs, confirming that their higher degrees of maturation compared with KIR^neg^ NK cells, also show a higher proportion of CD56^br^ and unCD56^dim^ NK cells.

Moreover, KIR^pos^ NK cells show a more active metabolic status than KIR^neg^ NK cells due to the enrichment of pathways associated with mitochondrial respiration. These results are consistent with recent studies uncovering the critical role of glycolysis and OxPhos in NK cell development, effector-functions, and generation of adaptive features (38). The upregulation of *CTP1A* and *TRAF6* in KIR^pos^ NK cells also suggests a preferential use of FAO to provide energy, exhibiting remarkable similarities to memory T cells (39, 40, 52). Finally, HCMV induces the downregulation of *SIGLEC-7* in KIR^pos^ NK cells, thus suggesting that the lack of this NKR might be associated with the acquisition of memory features (20).

The herein documented epigenetic reprogramming of KIR^pos^ NK cells highlights once more the great impact of HCMV in shaping “adaptive traits” in human NK cells by inducing the deregulation of zinc finger proteins (i.e., *ZBTB16*, *ZBTB32*, *ZBTB38*) in KIR^pos^ NK cells from R compared with both their KIR^neg^ counterparts in R and KIR^pos^ NK cells from NR. Of note, the reduction of *ZBTB16*/PLZF is consistent with the consequent decrease of *FCER1G*, *SYK*, and cytokine receptors observed in KIR^pos^ NK cells from R (27, 28). As a result of gene hypomethylation, KIR^pos^ NK cells show a higher *IFNG* transcript level in R compared with NR (29, 53). Furthermore, Gene Set Enrichment Analysis (GSEA) revealed that multiple pathways linked to IFN-γ signaling are specifically enriched in KIR^pos^ NK cells of R (29).

Although both flow cytometry data and transcriptional profiles clearly depicted the adaptive traits of KIR^pos^ NK cells from R, their molecular fingerprints reveal a state of metabolic/cellular exhaustion that we did not observe in NR. Indeed, our data showed the downregulation in KIR^pos^ NK cells from R of several pathways associated with cell migration and the cell cycle, while *NK cell-mediated cytotoxicity* pathways were enriched in KIR^pos^ NK cells from NR. In line with these results, our functional experiments showed an impaired ability of adaptive KIR^pos^ NK cells from R to produce IFN-γ compared with NR at 8–12 months after the transplant. In this context, a recent work showed that chronic stimulations of adaptive NK cells impairs their effector-functions and induces the expression of PD-1 and LAG-3 checkpoint inhibitors (33). In fact, and similar to what was reported for exhausted T cells (42), our results showed that KIR^pos^ NK cells from R are characterized by increased levels of *PDCD1* and *LAG3* compared with their counterparts in NR or to KIR^neg^ NK cells from R. Our masking experiments confirmed that the blocking of either PD-1 or LAG-3 increases the ability of NK cells from R to produce IFN-γ, although never reaching the levels of NK cells from NR, and to efficiently control HCMV infection in vitro. Taken together, these data highlight a remarkable dysfunction of adaptive NK cells due to HCMV-induced NK cell exhaustion and expression of inhibitory checkpoints. This functional impairment is reflected by a higher probability of experiencing a GVHD in patients with KIR^pos^ NK cell expansion. Further investigations are required to understand how to properly expand more efficient and less exhausted adaptive NK cells in an in vivo human setting of viral rechallenge.

In summary, the present study sheds new light on the physiopathology of adaptive NK cells in response to chronic and multiple HCMV infections and paves the way to develop an immune-therapeutic and NK cell-based approach to improve the control of opportunistic viral infections in h-HSCT.

## Methods

### Patient recruitment.

In this study, we enrolled 48 patients (Table 1) affected by hematologic malignancies and treated according to our published h-HSCT protocol (4). Patients were recruited at the Hematology and Bone Marrow Transplant Unit, Humanitas Cancer Center, Humanitas Research Hospital, Rozzano, Milan, Italy. Blood samples were collected from both donors and recipients before the transplantation, and from the recipients every month until 1 year after h-HSCT. The PBMCs were isolated from patients and relative HDs (54).

According to our institutional protocol, patients who had undergone h-HSCT were monitored for the HCMV infection/reactivation by assessing the HCMV viral load through real-time PCR (CMV R-GENE, Argene, Biomérieux) in the PB of both donor and recipients before and every week after the transplant to identify those patients experiencing HCMV infection/reactivation. Patients showing a viral load higher than 2,000 IU/mL, at least at one time point after the transplant, were considered as HCMV-reactivated and were treated with preemptive therapy (Foscarnet 90 mg/kg or Ganciclovir 5 mg/kg and/or Valganciclovir, twice a day for 2 weeks) when the viral load exceeded 4,000 IU/mL.

### Polychromatic flow cytometry and cell sorting.

All the flow cytometry experiments were performed in batch on frozen cells to minimize variability. Frozen cells were thawed and stained as we previously described (10). The anti-human monoclonal antibodies (mAbs) used for the analysis of NK cells of both patients who had undergone h-HSCT and relative HDs are listed in Supplemental Table 1.

Samples were acquired at BD FACSymphony A5 flow cytometer for the analysis of NK cell phenotype, while FACS experiments were performed at BD FACS Aria III (both from BD Bioscience).

### Unsupervised high-dimensional flow cytometry data analysis.

Flow Cytometry Standard (FCS) 3.0 files were imported into FlowJo software (version 9.9.6., TreeStar), and analyzed by standard manual gating strategy in order to remove debris, doublets, and dead cells, as well as to identify NK cells (Supplemental Figure 1A). Two thousand NK cells per sample were imported in FlowJo (version 10.2.0), biexponentially transformed and exported for further analysis in Python (version 3.7.3) using a custom pipeline with PhenoGraph as methods for clustering (http://github.com/luglilab/Cytophenograph, where we modified the linux-community and the core.py script of the PhenoGraph package in order to fix the seed to “123456”). Each sample was labeled with a barcode for further identification, converted into comma separated (CSV) files and concatenated in a single matrix by using the “concat” function of the “pandas” package.

The K-value, indicating the number of nearest neighbors identified in the first iteration of the algorithm, was set at 45. The data were then reorganized and saved as new CSV files, 1 for each cluster, that were further analyzed in FlowJo (version 10.2.0) to determine the frequency of positive cells for each marker and visualized in a heatmap (Figure 2A). Clusters representing less than 0.5% were excluded from the following analysis. Subsequent metaclustering of frequencies was performed using the gplots R package (35). Finally, PhenoGraph clusters were visualized using the UMAP technique.

### RNA isolation and retrotranscription.

The highly pure (> 95%) FACS-sorted NK cell subpopulations were lysed in 49 μL of RLT buffer (Qiagen) containing 1 μL of RNAse inhibitor (Thermo Fisher Scientific) and cryopreserved at –80°C.

Total RNA was purified with the MicroRNAeasy KitTM with DNAse (Qiagen), according to the manufacturer’s instructions and quantified by a Nanodrop 2000 (Thermo Fisher Scientific). Purified RNA was reverse-transcribed using a High Capacity cDNA Reverse transcription Kit (Thermo Fisher Scientific).

### Real-time PCR.

Real-time PCR was carried out on 7900HT Fast Real-Time PCR system (Thermo Fisher Scientific) using the TaqMan Universal PCR Master Mix and the following primers and probes: *ZBTB16* (PLZF, Hs00232313_m1), *SH2D1B* (EAT-2, Hs01592483_m1), *SYK* (SIK, Hs00895377_m1), *FCER1* (FcεRγ, Hs00175408_m1), and *GAPDH* (GAPDH, Hs02758991_g1) (all from Thermo Fisher Scientific). Gene expression levels were normalized to *GAPDH* control and calculated using the 2^–ΔΔCt^ method.

### RNA-Seq.

After RNA extraction, a RNA quality control was performed with the 4200 Tape Station system (Agilent). Only RNAs having an RNA Integrity Number (RIN) greater than 6 were used for library preparations. Total-RNA-Seq library preparation was performed starting from 0.5 ng of total-RNA with the SMART-Seq Stranded Kit (Clontech-Takara). Libraries obtained were qualitatively assessed by using TapeStation 4200 (Agilent) and quantified by Qubit Fluorimeter (Thermo Fisher Scientific). Afterward, they were multiplexed in equimolar pools and sequenced on a NextSeq-550 Illumina Platform generating at least 60 million 75bp-paired-end reads per sample.

RNA-Seq data are available at GEO under accession number GSE160362.

### RNA-Seq data analysis.

After quality control, reads were aligned to the reference genome (GRCh38.p12) using the STAR aligner with default parameters (version 2.7.0). Gene-based read counts were then obtained using feature count (55) module and GENCODE v29.gtf annotation (56). The read counts were imported into R statistical software, and differential gene expression analysis was performed using the Deseq2 package (57). Raw read counts were normalized using the Relative Log Expression method (RLE), and genes with low counts were filtered out by setting the parameter “independence filtering” as True. *P* values were adjusted using the Benjamini-Hochberg method.

### Differential gene expression analysis.

Genes were considered differentially expressed with FDR less than 0.05, and the PCA plot was generated using the plotPCA function of the Deseq2 package.

Functional enrichment analysis was performed using GSEA (version 3.0) software (Broad Institute of MIT) and gene list-ranked based on log_2_-fold changes. The gene set enrichment analysis was conducted in the preranked mode with scoring scheme “classic” and 1,000 permutations. The maximum gene set size was fixed at 5,000 genes and the minimum size was fixed at 10 genes. The gene signature was retrieved from the H (hallmark) and C2 (curated) collections (h.all.v6.2.symbols.gmt) of the Molecular Signatures Database (MSigDB v6.2). For GSEA only pathways with a |NES| greater than or equal to 1.8 and an FDR *q* value less than or equal to 0.05 were selected (58).

Gene correlation analysis and module detection were computed with the WGCNA R package (version 1.69; ref. 59).

### Functional assays.

The EBV-transformed human B cell line 721.221.G, transfected to express HLA-E, was a gift from Miguel López-Botet (Hospital del Mar Medical Research Institute, University Pompeu Fabra, Barcelona, Spain; ref. 60). 721.221.G cells were cultured in Roswell Park Memorial Institute (RPMI) medium supplemented with 10% FBS (Lonza), 1% Penicillin-Streptomycin (Invitrogen), 1% Ultra Glutamine (Lonza) at a density of 3 × 10^5^ cells/mL in a humidified atmosphere at 37°C with 5% CO_2_.

The EndoGRO HUVECs (MilliporeSigma) were cultured in endothelial cell growth medium-2 (EGM-2) (Lonza) supplemented with 5% FBS, 1% Penicillin-Streptomycin, and 1% Ultra Glutamine in cell flasks precoated with Collagen I (Corning) at a density of 10^5^ to 10^6^ cells/mL in a humidified atmosphere at 37°C with 5% CO_2_. The HUVECs were treated with IFN-γ (100 ng/mL) for 3 days to induce the expression of PD-L1 and HLA-II and further used as target cells in functional assays.

The PBMCs were thawed and incubated overnight in RPMI complete medium supplemented with rhIL-2 (100 IU/mL) and rhIL-12 (100 IU/mL) at 37°C in 5% CO_2_ (45). The following day, PBMCs were seeded at 1 × 10^6^ cells/mL in RPMI complete medium with rhIL-2 (100 U/mL) in the presence of HLA-E^pos^ 721.221.G target cells at PBMC/target ratio of 5:1 in the presence of Golgi Plug (BD Bioscience).

Alternatively, PBMCs were incubated in the presence of blocking mAbs (10 μg/mL): α–PD-1 (EH12.27H7; 329926; BioLegend) or α–LAG-3 (17B4; AG-20B-0012PF; AdipoGen) in 5% CO_2_ humidified atmosphere at 37°C for 30 minutes (42). Subsequently, PBMCs were seeded at 1 × 10^6^ cells/mL in RPMI complete medium with rhIL-2 (100 U/mL) and cultured on IFN-γ stimulated HUVECs at a PBMCs/target ratio of 6:1 in the presence of Golgi Plug.

After 4 hours of coculture, intracellular IFN-γ expression on NK cells was assessed by flow cytometry (Supplemental Table 1).

### In vitro HCMV infections.

Infection of HUVECs, previously incubated with IFN-γ for 3 days as detailed above, was performed at multiplicity of infection (MOI) of 1 by using the bacterial artificial chromosome (BAC) clone of the viral strain TB40/E expressing enhanced green fluorescent protein (EGFP; refs. 61, 62).

In parallel, KIR^pos^ NK cells FACS-sorted from PBMCs of NR and R recipients at 8–12 months after h-HSCT and left overnight in RPMI complete medium supplemented with rhIL-2 (100 IU/mL) and rhIL-12 (100 IU/mL) at 37°C in 5% CO_2_. The next day, purified KIR^pos^ NK cells (150,000 cells/mL) were incubated or not in the presence of α–PD-1 or α–LAG-3 blocking antibodies and cocultured for 48 hours with infected HUVECs in RPMI complete medium supplemented with rhIL-2 (200 IU/mL). The images were acquired to the DMi8 Wide-Field Microscope (Leica). Four representative fields for each well were analyzed and fluorescent-infected HUVECs were counted by using ImageJ software (NIH).

### Statistics.

Statistical analysis was performed using GraphPad PRISM (version 7.0) and R statistical software. An unpaired, 2-tailed Student’s *t* test was used to compare the different variables among samples. A paired, 2-tailed Student’s *t* test was used to compare the different variables within the same sample. One-way ANOVA with Bonferroni correction was used to perform multiple comparisons. Results are shown as the mean ± standard deviation (SD) or as the mean ± SEM when repeated sampling was considered.

Categorical variables were expressed as numbers, and comparisons between groups were performed using a χ^2^ test. Cumulative incidences of acute and chronic GVHD were constructed in the competing risks framework considering death without GVHD as competing events. The difference between cumulative incidence curves in the presence of a competing risk was tested using the Gray method. OS was estimated using the Kaplan-Meier method and the log-rank test was used for between-groups comparisons.

*P* values are two-sided or one-sided and were considered significant when *P ≤* 0.05 (* *P <* 0.05, ** *P <* 0.01, *** *P <* 0.001).

### Study approval.

This study has been approved by the Institutional Review Boards of the Clinical and Research Institute Humanitas (Approval 24/18). All patients and donors signed a written informed consent form in accordance with the Declaration of Helsinki.

## Author contributions

CDV, EL, and DM ideated the study. EZ, MC, AR, and JJPVB performed the experiments. FDP and GS processed the blood samples. SP and ST performed the bioinformatic analyses. CP performed and supervised the RNA-Seq experiments. JM, CDP, BS, SB, AS, and LC recruited the and collected biological specimens. RM assessed the HCMV serostatus of the patients who underwent h-HSCT. VTKLT and MT provided the BAC clone of the TB40/E-EGFP and contributed to perform the in vitro infection experiments. EZ, CDV, DM, and EM interpreted the data. EZ, CDV, and MC performed the data analyses and drew the figures. EZ, CDV, and DM wrote the article. All authors read and approved the final manuscript.

## Supplementary Material

Supplemental data

## Figures and Tables

**Table 2:**
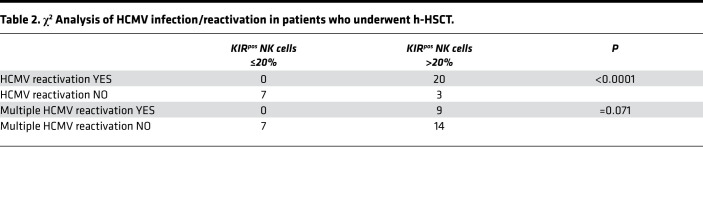
χ^2^ Analysis of HCMV infection/reactivation in patients who underwent h-HSCT.

**Table 1:**
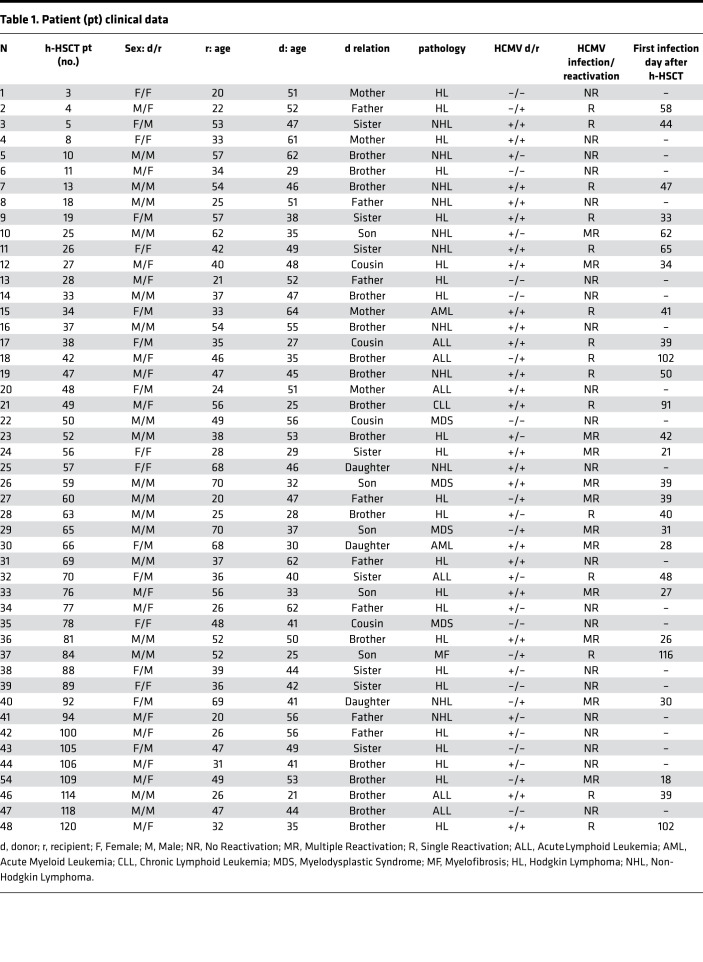
Patient (pt) clinical data

## Version 1 05/18/2021

In-Press Preview

## Version 2 06/22/2021

Electronic publication
