# Supplementary material for: Single-cell profiling identifies impaired adaptive NK cells expanded after HCMV reactivation in haploidentical HSCT
Source: JCI Insight. 2021 Jun 22;6(12):e146973. doi: 10.1172/jci.insight.146973 (PMC8262468; doi:10.1172/jci.insight.146973)
Supplement: Supplemental data [file jciinsight-6-146973-s022.pdf]

Single-cell profiling identifies impaired adaptive NK cells expanded after HCMV reactivation in haploidentical-HSCT

Elisa Zaghi[1], Michela Calvi[1,2], Simone Puccio[3], Gianmarco Spata[1], Sara Terzoli[1], Clelia Peano[4], Alessandra Roberto[3], Federica De Paoli[3], Jasper J.P. van Beek[3], Jacopo Mariotti[5], Chiara De Philippis[5], Barbara Sarina[5], Rossana Mineri[6], Stefania Bramanti[5], Armando Santoro[5], Vu Thuy Khanh Le-Trilling[7], Mirko Trilling[7], Emanuela Marcenaro[8], Luca Castagna[5], Clara Di Vito[1,2†], Enrico Lugli[3,9] and Domenico Mavilio[1,2†*]*

[1]*Unit of Clinical and Experimental Immunology, IRCCS Humanitas Research Hospital, Rozzano, Milan, Italy;*
[2]*BIOMETRA, Università degli Studi di Milano, Milan, Italy;*
[3]*Laboratory of Translational Immunology, IRCCS Humanitas Research Hospital, Rozzano, Milan, Italy;*
[4]*Institute of Genetic and Biomedical Research, UoS Milan, National Research Council, and Genomic Unit, IRCCS Humanitas Research Hospital, Italy;*
[5]*Bone Marrow Transplant Unit, IRCCS Humanitas Research Hospital, Rozzano, Milan, Italy;*
[6]*Molecular Biology Section, Clinical Investigation Laboratory, IRCCS Humanitas Research Hospital, Rozzano, Italy;*
[7]*Institute for Virology, University Hospital Essen, University Duisburg-Essen, Essen, Germany;*
[8]*Department of Experimental Medicine, University of Genoa, Genoa, Italy;*
[9]*Flow Cytometry Core, IRCCS Humanitas Research Hospital, Rozzano, Milan, Italy.*

[†]*These authors contributed equally to this work*

*** Corresponding authors:**
Domenico Mavilio, M.D., Ph.D.
Unit of Clinical and Experimental Immunology
Department of Medical Biotechnologies and Translational Medicine
University of Milan School of Medicine
IRCCS Humanitas Research Hospital
Via Alessandro Manzoni, 113, Rozzano (Milan), Italy.
Phone: +39-02.8224.5157 FAX: +39-02.8224.5191
e-mail: domenico.mavilio@unimi.it

Clara Di Vito, Ph.D.
Unit of Clinical and Experimental Immunology
Department of Medical Biotechnologies and Translational Medicine
University of Milan
IRCCS Humanitas Research Hospital
Via Alessandro Manzoni, 113, Rozzano (Milan), Italy.
Phone: +39 02 8224 5220
e-mail: clara.di_vito@humanitasresearch.it

Supplemental data

Supplementary Figures

Fig. S1. NK cell gating strategy

Fig. S2. NK cell marker dynamic expression in MR h-HSCT patients

Fig. S3. NK cell subset distribution in FACS-sorted KIR^{pos} and KIR^{neg} NK cells

Fig. S4. KIR^{pos} NK cells from R show a different expression profile compared to KIR^{neg} NK cells from R and KIR^{pos} NK cells from NR

Fig. S5. HCMV-driven transcriptional changes in KIR^{pos} NK cell from R

Fig. S6. IFN-γ inducible expression of PD-L1 and HLA-II on HUVEC

Fig. S7. KIR^{pos} NK cell frequency does not correlate with the OS

Supplementary Table

Table S1. List of antibodies used in flow cytometry panels

Supplementary Figures

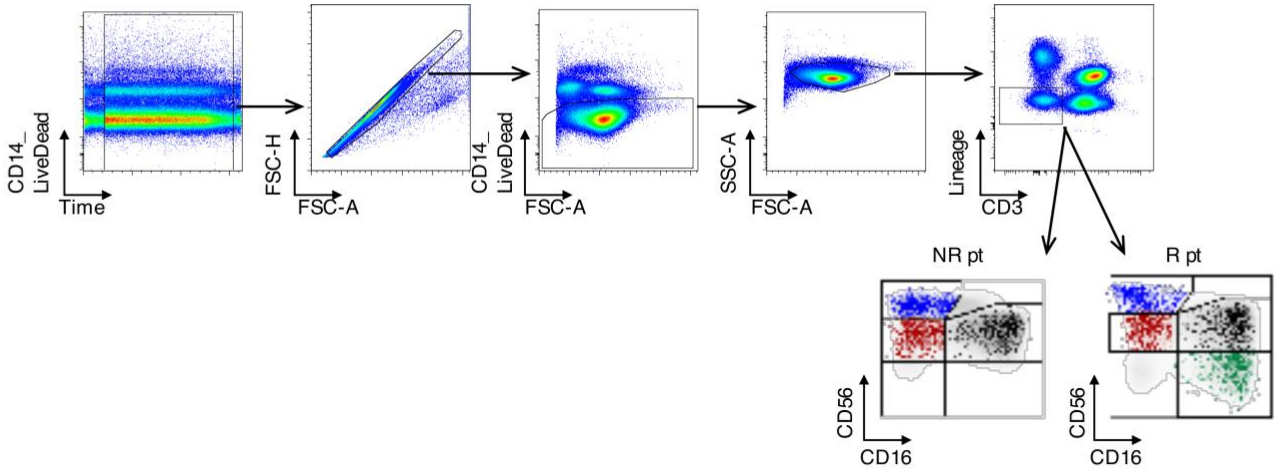

Fig. S1. NK cell gating strategy

Flow cytometry dot plots showing the gating strategy used to identify NK cells within $CD14^{neg}/CD3^{neg}$ and $Lineage^{neg}$ (CD4, CD15, CD20, CD19, CD33, CD34, CD203c, FCεRI) viable lymphocytes and the NK cell subset distribution on the basis of their CD56 and CD16 surface expression from representative patients either experiencing (R, right, pt#27) or not (NR, left, pt#3) HCMV infection/reactivation at 10-12 months after h-HSCT. $CD56^{bright}/CD16^{neg}$ ($CD56^{br}$) NK cells are depicted in blue, $CD56^{dim}/CD16^{pos}$ ($CD56^{dim}$) NK cells are depicted in black, unconventional $CD56^{dim}/CD16^{neg}$ ($unCD56^{dim}$) are depicted in red and $CD56^{neg}/CD16^{pos}$ ($CD56^{neg}$) NK cells are depicted in green.

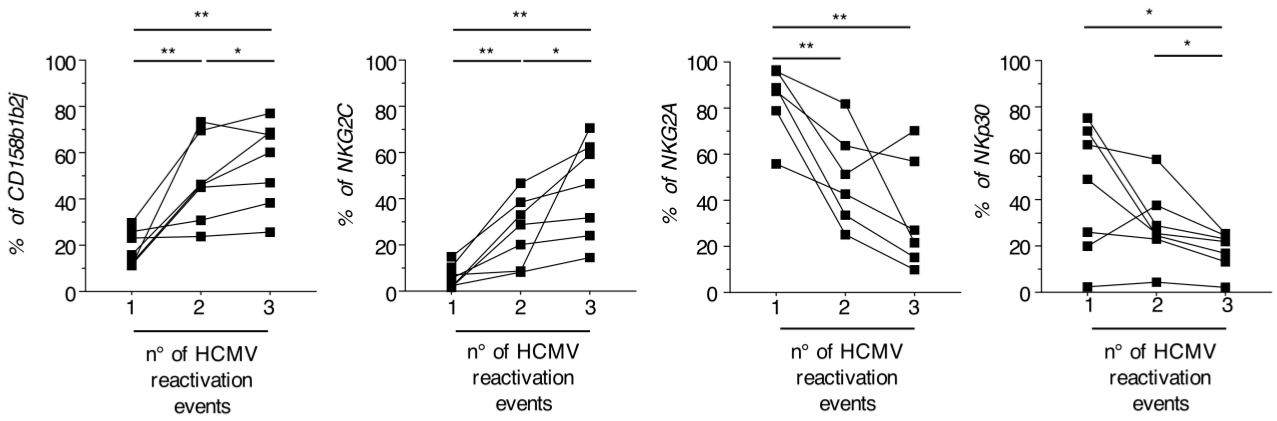

Fig. S2. NK cell marker dynamic expression in MR h-HSCT patients
Summary statistical graphs showing the frequencies (%) of CD158b1b2j, NKG2C, NKG2A, and NKp30 on total NK cells on recipients (n=7) experiencing HCMV multiple reactivation events (n°=1, 2, and 3) after h-HSCT. Blood samples of MR were analyzed at the first available time point (range: 0-21 days) after the pick of viremia defining HCMV reactivation events. Paired t-test.

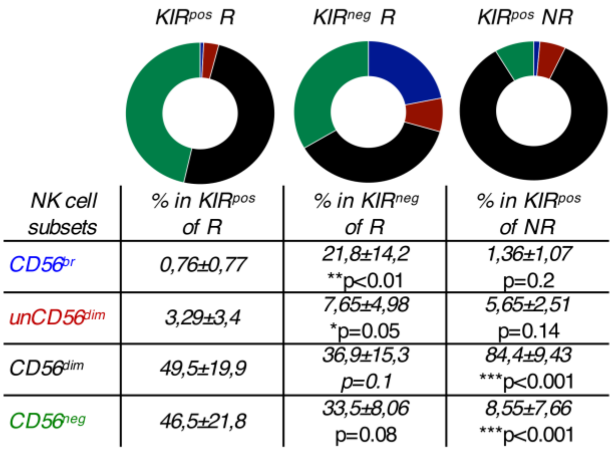

NK cell subsets	% in KIR^{pos} of R	% in KIR^{neg} of R	% in KIR^{pos} of NR
CD56^{br}	0,76±0,77	21,8±14,2<br>**p<0.01	1,36±1,07<br>p=0.2
unCD56^{dim}	3,29±3,4	7,65±4,98<br>*p=0.05	5,65±2,51<br>p=0.14
CD56^{dim}	49,5±19,9	36,9±15,3<br>p=0.1	84,4±9,43<br>***p<0.001
CD56^{neg}	46,5±21,8	33,5±8,06<br>p=0.08	8,55±7,66<br>***p<0.001

Fig. S3. NK cell subset distribution in FACS-sorted KIR^{pos} and KIR^{neg} NK cells
Pie charts depicted the NK cell subset distribution within the FACS-sorted KIR^{pos} and KIR^{neg} NK cells (n=7) recipients at 7-12 months after h-HSCT. The relative frequencies of NK cell subsets (mean ± SD) and p-values vs KIR^{pos} NK cells of R are reported. Paired t-test vs KIR^{neg} of NR; Unpaired t-test vs KIR^{pos} R.

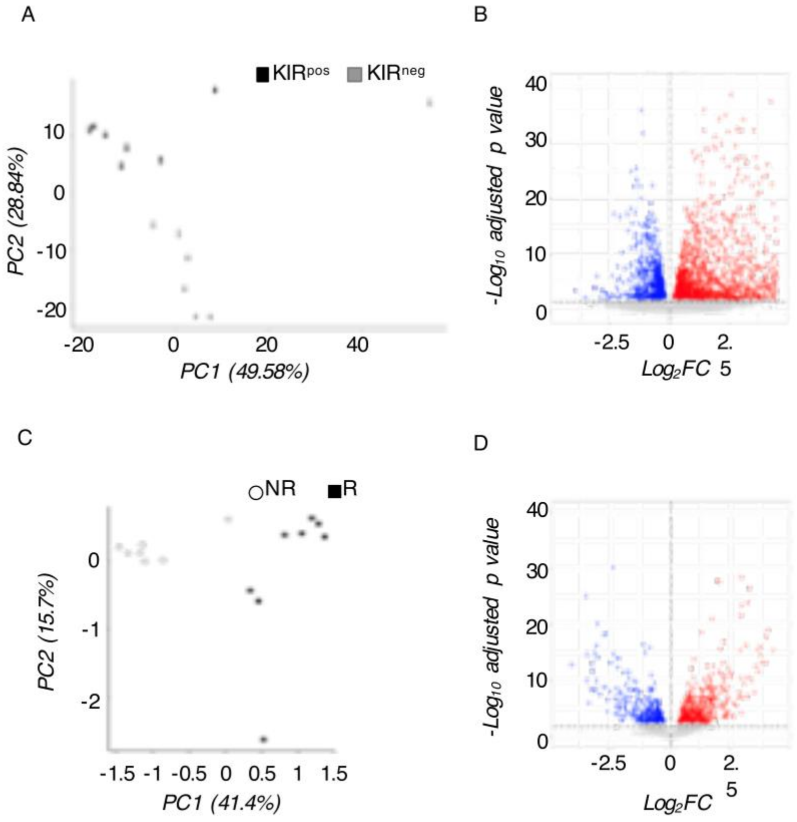

Fig. S4. KIR^{pos} NK cells from R show a different expression profile compared to KIR^{neg} NK cells from R and KIR^{pos} NK cells from NR

(A) PCA scatter plots showing the bi-dimensional distribution of in KIR^{neg} (■) and KIR^{pos} (■) FACS-sorted NK cells from R recipients after 7-12 months after h-HSCT (n=7). **(B)** Volcano plot representing the deregulated genes between KIR^{neg} and KIR^{pos} FACS-sorted NK cells. The differentially upregulated (red), downregulated (blue), and stable (gray) genes in KIR^{pos} NK cells are depicted. **(C)** PCA scatter plots of gene expression in KIR^{pos} NK cells FACS-sorted from NR (○; n= 8) and R (■; n=7) after 7-12 months after h-HSCT. **(D)** Volcano plot representing the deregulated genes between KIR^{pos} FACS-sorted NK cells from R and NR. The differentially upregulated (red), downregulated (blue), and stable (gray) genes in KIR^{pos} NK cells from NR are depicted.

A

GENE ONTOLOGY	NES	FDR q-value
GO_REGULATION_OF_CELLULAR_COMPONENT_MOVEMENT	2,80	0,002074
GO_POSITIVE_REGULATION_OF_LOCOMOTION	2,51	0,013466
GO_POSITIVE_REGULATION_OF_CHEMOTAXIS	2,39	0,025039
GO_REGULATION_OF_ACTIN_FILAMENT_BUNDLE_ASSEMBLY	2,38	0,025315
GO_TAXIS	2,31	0,031714
GO_REGULATION_OF_ACTIN_FILAMENT_BASED_PROCESS	2,26	0,031429
GO_REGULATION_OF_CHEMOTAXIS	2,24	0,032622
GO_CELL_CHEMOTAXIS	2,21	0,036473
GO_LEUKOCYTE_MIGRATION	2,21	0,036062
GO_ACTIN_FILAMENT_BASED_PROCESS	2,14	0,047366
GO_REGULATION_OF_LAMELLIPODIUM_ORGANIZATION	2,15	0,04758

B

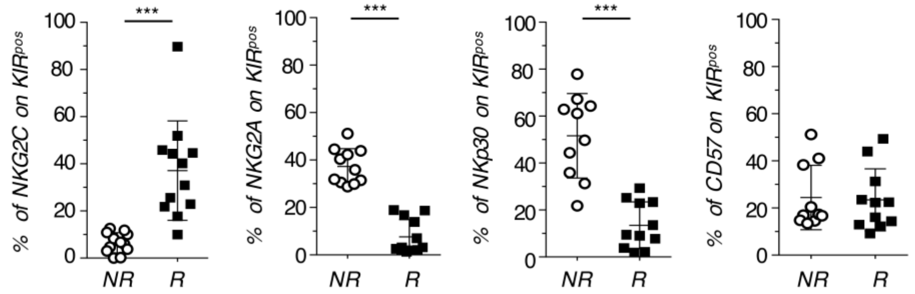

C

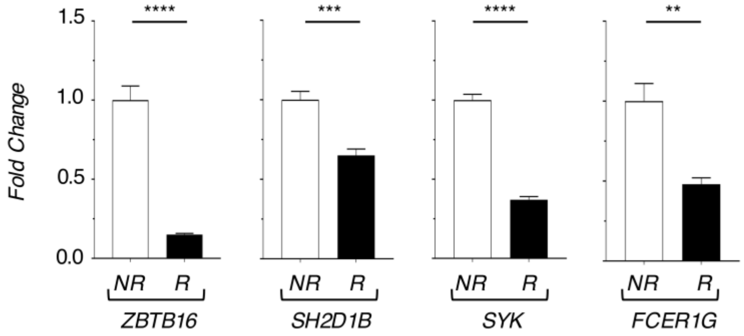

Fig. S5. HCMV-driven transcriptional changes in KIR^{pos} NK cell from R

(A) NES and FDR of GO signature involved in cell migration enriched in NR vs R. **(B)** Summary statistical graphs showing the expression (%; mean ± SD) of NKG2C, NKG2A, NKp30, and CD57 on NK cells in NR (○, n=12) and R (■, n=12) patients at 7-12 months post-h-HSCT. Unpaired t-test. **(C)** Summary statistical graphs showing the expression levels of *ZBTB16*/PLZF, *SH2D1B*/EAT-2, *SYK*/SYK, and *FCER1G*/FcεRγ genes assessed by semi-quantitative real-time PCR on FACS-sorted KIR^{pos} NK cells in NR (○; n=6) and in R (■; n=6) patients at 8-12 months post-h-HSCT. Each sample was analyzed in triplicate. Gene expression was normalized on KIR^{pos} NK cells of NR h-HSCT patients. Unpaired t-test.

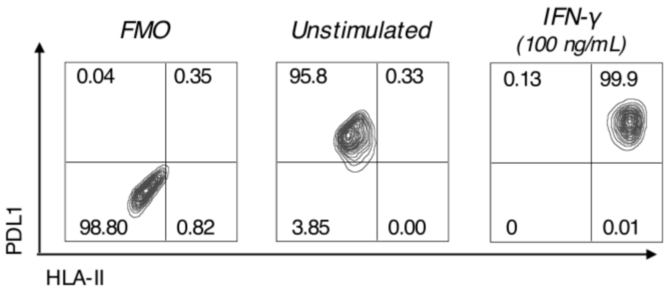

Fig. S6. IFN-γ inducible expression of PD-L1 and HLA-II on HUVEC
Representative flow cytometry contour plots showing the surface expression of PD-L1 and HLA-II on HUVEC either in the presence or in the absence of stimulation with IFN-γ (100 ng/mL) for 72 hours. Fluorescence minus one (FMO) was used to set the gate on positive population.

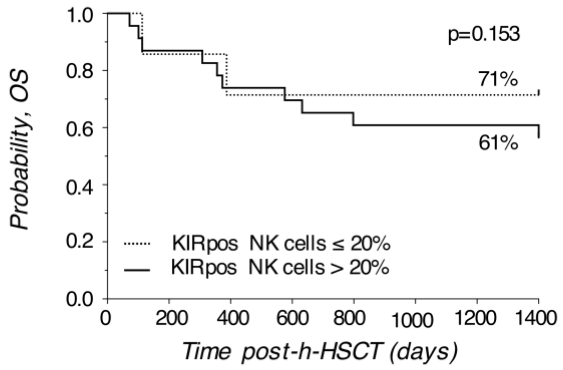

Figure S7. KIR^{pos} NK cell frequency does not correlate with the OS
Kaplan-Meier curve depicting the 4-years OS in h-HSCT patients subdivided based on the KIR^{pos} NK cell frequencies (KIR^{pos} NK cells <20%, n=7, dotted line; KIR^{pos} NK cells >20%, n=19, solid line).

Table S1. List of antibodies used in flow cytometry panels

Antibody (mAb)	Clone	Fluorochrome	Company
Anti-CD4	13B8.2	FITC	Beckman Coulter
Anti-CD15	80H5	FITC	Beckman Coulter
Anti-CD20	2H7	FITC	BioLegend
Anti-CD33	HIM3-4	FITC	BioLegend
Anti-CD34	561	FITC	BioLegend
Anti-CD203c	NP4D6	FITC	BioLegend
Anti-FCeRI	AER37	FITC	BioLegend
Anti-CD19	SJ25C1	FITC	BD Biosciences
Anti-CD14	M5E2	BV510	BioLegend
Anti-CD3	UCHT1	BUV661	BD Biosciences
Anti-CD16	3G8	BUV737	BD Biosciences
Anti-CD16	3G8	PE-Cy7	BD Biosciences
Anti-CD56	NCAM16-2	BUV563	BD Biosciences
Anti-NKG2A	REA110	PE-Vio770	Miltenyi
Anti-NKG2C	134591	AF700	R&D
Anti-NKG2C	134591	BV650	BD Biosciences
Anti-CD57	REA769	PE-Vio615	Miltenyi
Anti-CD8	RPA-T8	BV650	BD Biosciences
Anti-CD158b1b2j	CHL	BUV395	BD Biosciences
Anti-CD158b1b2j	GL183	PE-Cy5.5	Beckman Coulter
Anti-CD158a	HP3E4	BV711	BD Biosciences
Anti-CD158e	DX9	APC	Miltenyi
Anti-CD158e1/e2	Z27.3.7	PE	Beckman Coulter
Anti-NKp30	Z25	PE-Cy5	Beckman Coulter
Anti-NKp46	9E2	BV605	BioLegend
Anti-PD1	PD1.3.1.3	APC	Miltenyi
Anti-PD1	EH12.1	BV711	BD Biosciences
Anti-CD127	eBIORDR5	APCeFluor780	eBiosciences
Anti-CRTh2	BM16	PerCP-Cy5.5	BioLegend
Anti-CD117	104D2	BV786	BioLegend
Anti-IFN-γ	B27	BUV395	BD Biosciences
Anti-PDL1	MIH1	PE-CF594	BD Biosciences
Anti-HLA-DR/DP/DQ	Tu39	FITC	BD Biosciences
